# Metabolomic profiles reveal physiological transitions required for long-distance avian migration

**DOI:** 10.1038/s41598-026-41603-2

**Published:** 2026-04-04

**Authors:** Jonathan Vergara-Amado, Pablo Alarcón, Rafael A. Burgos, Juan G. Navedo, Josefina Gutiérrez, Claudio Verdugo

**Affiliations:** 1 Ecology of Infectious Diseases Lab, Instituto de Patología Animal, Facultad de Ciencias Veterinarias, Universidad Austral de Chile, Valdivia, Chile; 2 Bird Ecology Lab, Instituto de Ciencias Marinas y Limnológicas, Universidad Austral de Chile, Valdivia, Chile; 3 Programa de Doctorado en Ciencias mención Ecología y Evolución, Escuela de Graduados, Facultad de Ciencias, Universidad Austral de Chile, Valdivia, Chile; 4 Instituto de Farmacología y Morfofisiología, Facultad de Ciencias Veterinarias, Universidad Austral de Chile, Valdivia, Chile; 5 Millennium Institute Biodiversity of Antarctic and Subantarctic Ecosystems (BASE), Santiago, Chile; 6 Estación Experimental Quempillén, Chiloé, Facultad de Ciencias, Universidad Austral de Chile, Valdivia, Chile; 7 Center for Surveillance and Evolution of Infectious Diseases, Facultad de Ciencias Veterinarias, Universidad Austral de Chile, Valdivia, Chile; 8 Ecology in the Anthropocene, Área de Zoología, Universidad de Extremadura, Badajoz, Spain

**Keywords:** Metabolomics, Migration, Non-breeding season, Long-distance migratory birds, Biochemistry, Physiology, Systems biology, Zoology

## Abstract

Long-distance migratory birds undergo profound physiological changes throughout their annual cycle, particularly during the non-breeding season, which includes periods of recovery, maintenance, and preparation for migration. Here, we used an untargeted gas chromatography–mass spectrometry (GC-MS) metabolomic approach to characterize plasma metabolite profiles in Hudsonian godwits (*Limosa haemastica*) across three distinct stages of the non-breeding season: *post-arrival*, *maintenance*, and *pre-departure*. We identified 141 metabolites associated with key metabolic pathways, including carbohydrate, amino acid, and lipid metabolism. Metabolomic profiles showed a progressive seasonal transition, with clear separation between *post-arrival* and *pre-departure* stages, and individuals displaying an intermediate metabolic profile during *maintenance*. *Post-arrival* individuals exhibited metabolite signatures linked to muscle repair, lipid catabolism, and oxidative stress recovery. In contrast, *pre-departure* individuals showed upregulation of metabolites involved in carbohydrate remodeling, unsaturated fatty acid biosynthesis, and steroid metabolism, reflecting physiological preparation for long-distance flight. Our results reveal a dynamic and stage-specific metabolic strategy that underscores the phenotypic flexibility of godwits during the non-breeding season. These findings provide new insights into the physiological mechanisms underlying endurance migration and the metabolic flexibility of migratory shorebirds.

**Supplementary Information:**

The online version contains supplementary material available at 10.1038/s41598-026-41603-2.

## Introduction

Animal migration is an adaptive strategy that enables species to respond to predictable seasonal fluctuations in resources and environmental conditions^1,2^. Long-distance migratory birds, in particular, undertake one of the most energetically demanding activities in the animal kingdom, often traveling over 10,000 km non-stop endurance flights^2,3^⁠. To meet the extraordinary energetic demands of migration, birds undergo rapid and profound physiological and behavioral modifications^1,4^⁠. These physiological adjustments enable birds to maintain a balance in their metabolism throughout the annual cycle, adapting to contrasting phenotypic profiles, in relatively short time, that reflect the varying demands of different stages of the annual cycle, such as the post-arrival recovery or the pre-departure fattening for migratory flight^5,6^. Upon arrival after migration, individuals must recover from the intense physical exertion of prolonged flight, activating mechanisms to repair tissues and mitigate oxidative damage, such as elevated plasma levels of non-enzymatic antioxidants^7,8^⁠. Conversely, prior to departure, birds undergo a period of fattening, characterized by hyperphagia, fat accumulation, and lean mass growth. This process significantly increases the body mass to meet the high-energy demands of migration^9,10^. The metabolic adjustments involved during this pre-migratory fattening stage include the enlargement and increased functional capacity of the gizzard, intestine, and liver to optimize nutrient processing and energy storage^11–14^, and behavioral modifications, including hyperphagia and altered locomotor activity rhythms^15^, that support this fueling period. Together, these physiological and behavioral changes shape the metabolic profiles of long-distance migratory birds. However, the mechanisms underlying the flexibility^16^ of these contrasting phenotypes remain poorly understood.

Experimental and field-based research focusing on physiological and metabolic changes during key phases of the annual cycle of migratory birds has revealed critical shifts in the metabolic pathways. For instance, during the breeding season, females exhibit elevated levels of plasma triglycerides, proteins, and cholesterol, reflecting increased energy acquisition and storage to support egg production and gonadal development, as observed in seabirds^17,18^. During stopover periods, migratory passerines adjust their plasma metabolome, showing elevated unsaturated fatty acids and glycolytic intermediates, indicating increased fatty acid oxidation and glycolysis to meet energy demands, alongside variations in glucose and free fatty acid levels^19,20^⁠. During migratory flights, experimental studies on a shorebird (*Calidris canutus*) have shown elevated plasma levels of free fatty acids and glycerol, signaling the mobilization of fat stores from adipose tissue to fuel sustained flight^21^⁠. In addition, β-hydroxybutyrate levels increase, reflecting a metabolic shift that spares glucose and proteins during endurance flight, while protein catabolism is observed through elevated uric acid levels^21^. These findings emphasize the complex metabolic adjustments that occur in long-distance migratory birds, with lipid-based energy mobilization being central to sustaining flight and reproduction. Nevertheless, important knowledge gaps remain, particularly about the metabolic strategies operating during the fattening phase prior to migration.

The non-breeding season of the annual cycle of migratory birds is crucial for understanding the depth of metabolic mechanisms acting on the phenotypes associated with different stages during the season, including post-migratory recovery and pre-migratory fattening. The non-breeding season corresponds to a longer period than the breeding season, allowing a better discrimination of the phenotypes in the absence of metabolic and physiological demands associated with mating and breeding^22^⁠. Additionally, this period is particularly marked by the physiological preparation for migratory flights which, in some species, are characterized by few stopovers, culminating in a fast and high time-constrained journey during the spring migration^23,24^. Thus, this journey imposes significant physiological challenges due to limited stopovers to refuel and high travel speed^25^⁠. Previous research in long-distance migratory shorebirds has highlighted a significant increase in total antioxidant capacity and the mobilization of lipid reserves, including fatty acids and ketone bodies, as well as protein breakdown products, like uric acid, to provide the necessary energy for long-duration flights^22–26^. These findings underscore the importance of investigating the dynamics of metabolic profiles throughout the non-breeding season, that can provide critical insights into the physiological adaptations employed during fattening phase required for the energetic challenges of a long-distance migratory flight.

Metabolomics offers a powerful, unbiased approach for characterizing the complete collection of metabolites found in a biological sample^27^. Since metabolites are highly sensitive to external (i.e., environmental) stimuli and internal signaling (i.e., physiological and intracellular), the metabolome serves as an indicator of an individual’s phenotype, reflecting ongoing biological processes^27,28^⁠. However, most studies on the transition of extreme phenotypes, such as the avian preparation for migration, have focused on specific targeted metabolites^22,29,30^⁠, with few adopting an unbiased and untargeted metabolomics approach^19^⁠ that could provide a more comprehensive understanding of the metabolic adjustments involved during critical stages of migration.

In this study, we employed a non-targeted metabolomics approach to assess metabolic changes associated with phenotypic stages during the non-breeding season of the annual cycle of the Hudsonian godwit (*Limosa haemastica*), a long-distance migratory shorebird with a well-known annual cycle^31–33^⁠. We compared metabolomes across three key stages: (1) *post-arrival*, two to six weeks after arrival at the non-breeding grounds; (2) *maintenance* period; and (3) *pre-departure*, at the end of the fueling-up stage just prior to northbound migration. We hypothesize that each of these stages associated with different phenotypes of the non-breeding season will exhibit distinct metabolomic composition, reflecting the physiological adjustments of long-distance migratory birds in response to the different energetic demands of *post-arrival*, *maintenance*, and *pre-departure* periods. Understanding the metabolomic profiles underlying the phenotypic plasticity associated with contrasting stages of the annual cycle of long-distance migratory birds may provide essential insights into the physiological mechanisms that govern their unique migratory performance.

## Results

### Seasonal dynamics of body condition

**Fig. 1:**
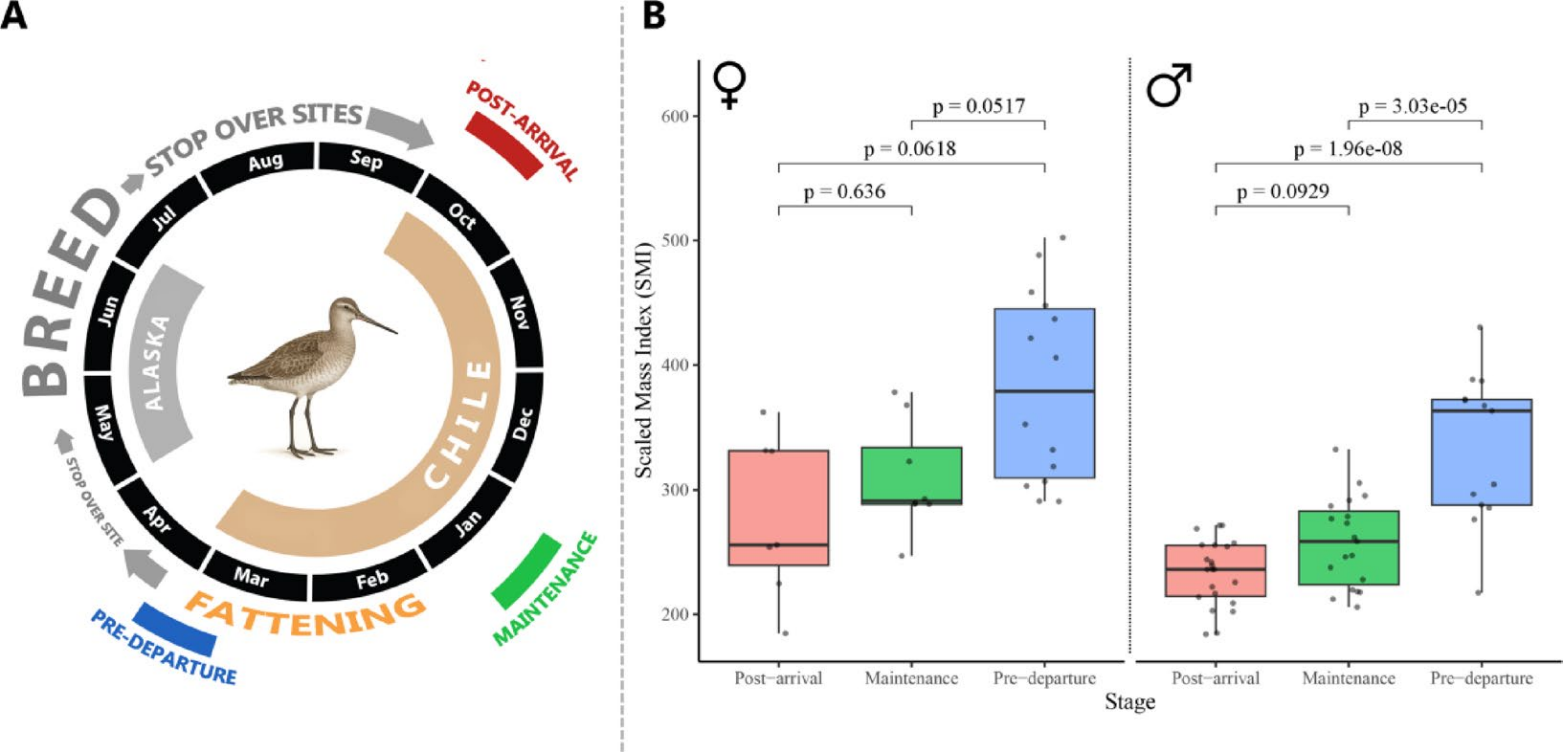
Seasonal changes in scaled mass index (SMI) of Hudsonian godwits (*Limosa haemastica)* during different stages of the non-breeding season. **(A)** Schematic representation of the annual cycle of *L. haemastica. *. (adapted from Conklin et al. (2015), using data from Navedo & Ruiz (2020) and Swift et al. (2020)), highlighting the three main stages during the non-breeding season: *post-arrival*, *maintenance*, and *pre-departure*. **(B)** SMI of female and male individuals across non-breeding stages. Horizontal bars indicate pairwise comparisons between stages based on estimated marginal means (EMMs) with Tukey-adjusted p-values. Significant differences (*p* < 0.05) are shown above the corresponding comparisons.

The scaled mass index (SMI) increased significantly across the non-breeding season in both sexes (females: 27.4%, *n* = 29, increase, *p* = 0.004; males: 30.3%, *n* = 55, increase, *p* < 0.0001), reflecting physiological preparation for migration. At *post-arrival* stage, the mean ± SD SMI was 277.8 ± 65.14 g for females and 233.17 ± 26.29 g for males, with fat scores being close to 0 (range: 0–1) for both sexes (*n* = 30). During *maintenance*, female SMI increased to 309.0 ± 44.46 (*p* = 0.051) and male SMI to 257.56 ± 35.94 (*p* < 0.001), with fat scores remained close to 0.0 (range: 0–1) (*n* = 27). By *pre-departure*, female SMI reached 382.64 ± 76.95 (*p* = 0.0062) and male SMI 334.59 ± 60.33 (*p* < 0.001), with fat scores averaging 5.1 and 5.5 (range: 3–7), respectively (*n* = 27; Fig. 1).

### Overall godwits metabolomic profile

GC-MS analysis qualified and quantified 141 plasma metabolites from 84 individuals. From those, 123 and 122 metabolites were annotated according to the PubChem and KEGG databases, respectively (Supplementary Fig. 1 A and 1B). Overall, the metabolomic profile, obtained from PubChem chemical classes, predominantly included organic acids (30.84%), carbohydrates (20.5%), and fatty acids (20.5%), which when mapped onto KEGG metabolic pathways corresponded to carbohydrates (33.6%), amino acid (30.33%), lipid (22.1%), and energy (5.74%) categories.

**Fig. 2:**
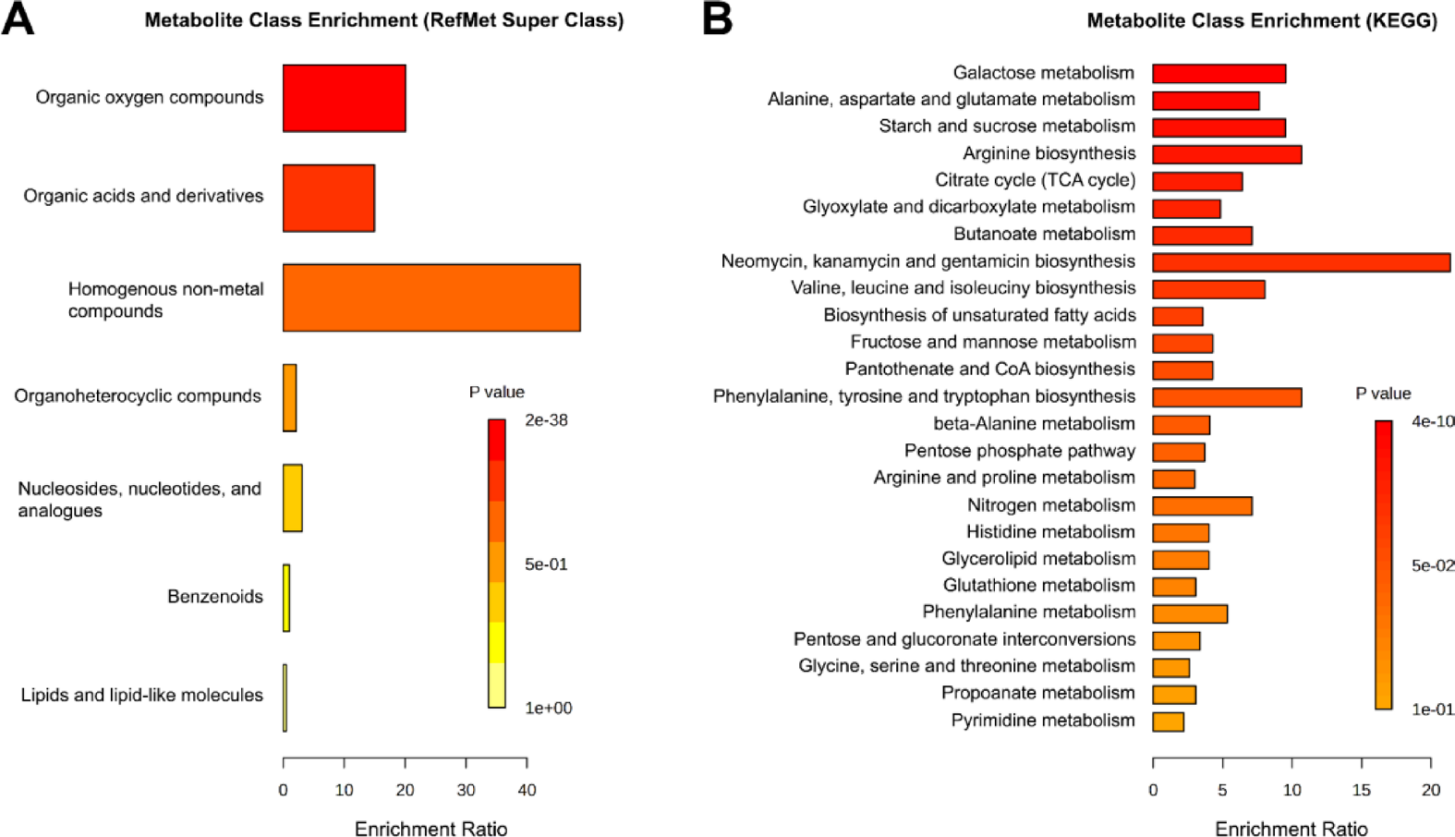
**(A)** Enrichment analysis output for chemical super-class of metabolomic profiles of *L. haemastica* during non-breeding season. Annotated metabolite list submitted to MetaboAnalyst for metabolite class enrichment analysis, compared against Metabolomics Workbench RefMet for chemical Super Class. Enrichment Ratio indicates the ratio of annotated features found to expected. **(B)** Enrichment analysis output for KEGG pathways associated with annotated plasma metabolites. Annotated metabolite lists were submitted to MetaboAnalyst v6.0 for metabolic pathway enrichment analysis based on KEGG metabolic pathways. Enrichment Ratio represents the ratio of observed metabolites within a given KEGG pathway relative to the expected number of observations. Pathway analysis based on KEGG database implemented in MetaboAnalyst.

Enrichment analyses using the RefMet super-class library revealed significant representation of organic oxygen compounds, organic acids and derivatives, homogeneous non-metal compounds, and nucleosides-nucleotides analogues (Fig. 2A). KEGG pathway analysis highlighted a significant enrichment of carbohydrate metabolism (including galactose metabolism, starch and sucrose metabolism, and citrate cycle), amino acid metabolism (arginine metabolism, alanine, aspartate, and glutamate metabolism) and lipid metabolism (biosynthesis of unsaturated fatty acids) (Fig. 2B).

### Metabolomic changes across the non-breeding season

**Fig. 3:**
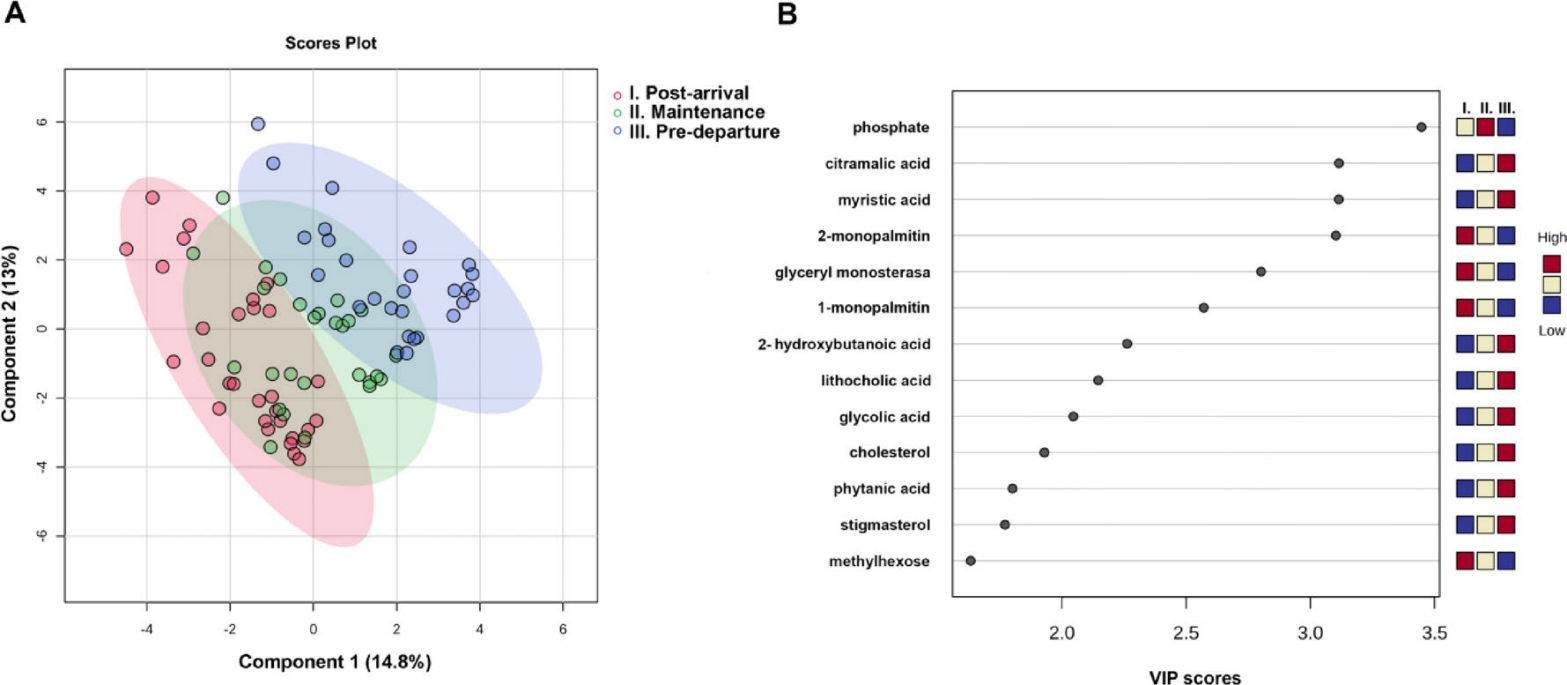
Partial Lead Squares Analysis and Variable importance in projection scores of plasma metabolites in Hudsonian godwits (*Limosa haemastica*) **(A)** Partial Lead Squares Analysis of annotated features from plasma samples of the godwits. **(B)** Variable importance in projection (VIP) scores of primary metabolites identified by partial least squares-discriminant analysis (PLS-DA). The 13 metabolites with the highest VIP scores are shown (> 1.6 VIP score). Colored boxes on the right represent the relative concentrations of metabolites in each group. The red and blue colors indicate that the metabolite level is increased or decreased (respectively) with respect to the mean of the relative metabolite abundance.

PLS-DA revealed clear clustering of samples according to each progressive stage of the non-breeding season (Fig. 3A). Despite overlapping considerably with the *maintenance* stage, *post-arrival* and *pre-departure* groups remained distinct from one another. The metabolomic profiles from individuals of the *post-arrival* stage were significantly different from the profiles of individuals from the *pre-departure* stage (Pr (> F) = 0.0004). In contrast, the profiles from individuals of the *maintenance* stage showed non-significant differences when compared to both *post-arrival* (Pr (> F) = 0.054) and *pre-departure* (Pr (> F) = 0.159) stages. Sex (Pr (> F) = 0.46) and sex-stage interaction (Pr (> F) = 0.18) did not significantly influence the metabolomic profiles of godwits.

Thirteen metabolites with VIP scores > 1.6 significantly contributed to the detected clustering (Fig. 3B). In the *post-arrival* stage, monoacylglycerol metabolites such as 2-monopalmitin, glyceryl monostearate, 1-monopalmitin, and methylhexose were significantly higher than in the *maintenance* and *pre-departure* stages. Conversely, metabolites involved in lipid-related pathways (e.g., myristic acid, cholesterol, lithocholic acid, stigmasterol), together with metabolites linked to central carbon metabolism (e.g., citramalic acid, glycolic acid, 2-hydroxybutanoic acid) were significantly higher in the *pre-departure* stage compared to the earlier stages. Phosphate was uniquely enriched during *maintenance*.

A two-way analysis of variance identified 49 metabolites that were significantly different across the stages after correction (Supplementary Fig. 2). No significant effects of sex or the sex-stage interaction were detected in the abundance of any of the metabolites (Supplementary Table 1). Individuals from the *post-arrival* stage showed 14 metabolites with higher abundance than in the *maintenance* and *pre-departure* stages. The *maintenance* stage showed seven metabolites with higher abundance than in the *post-arrival* and *pre-departure* stages, while four metabolites were significantly more abundant in both *maintenance* and *pre-departure* stages compared to the *post-arrival* stage. Finally, individuals from the *pre-departure* stage showed 24 metabolites with higher abundance than in the *maintenance* and/or *post-arrival* stages.

### Hierarchical clustering of metabolites across stages

**Fig. 4:**
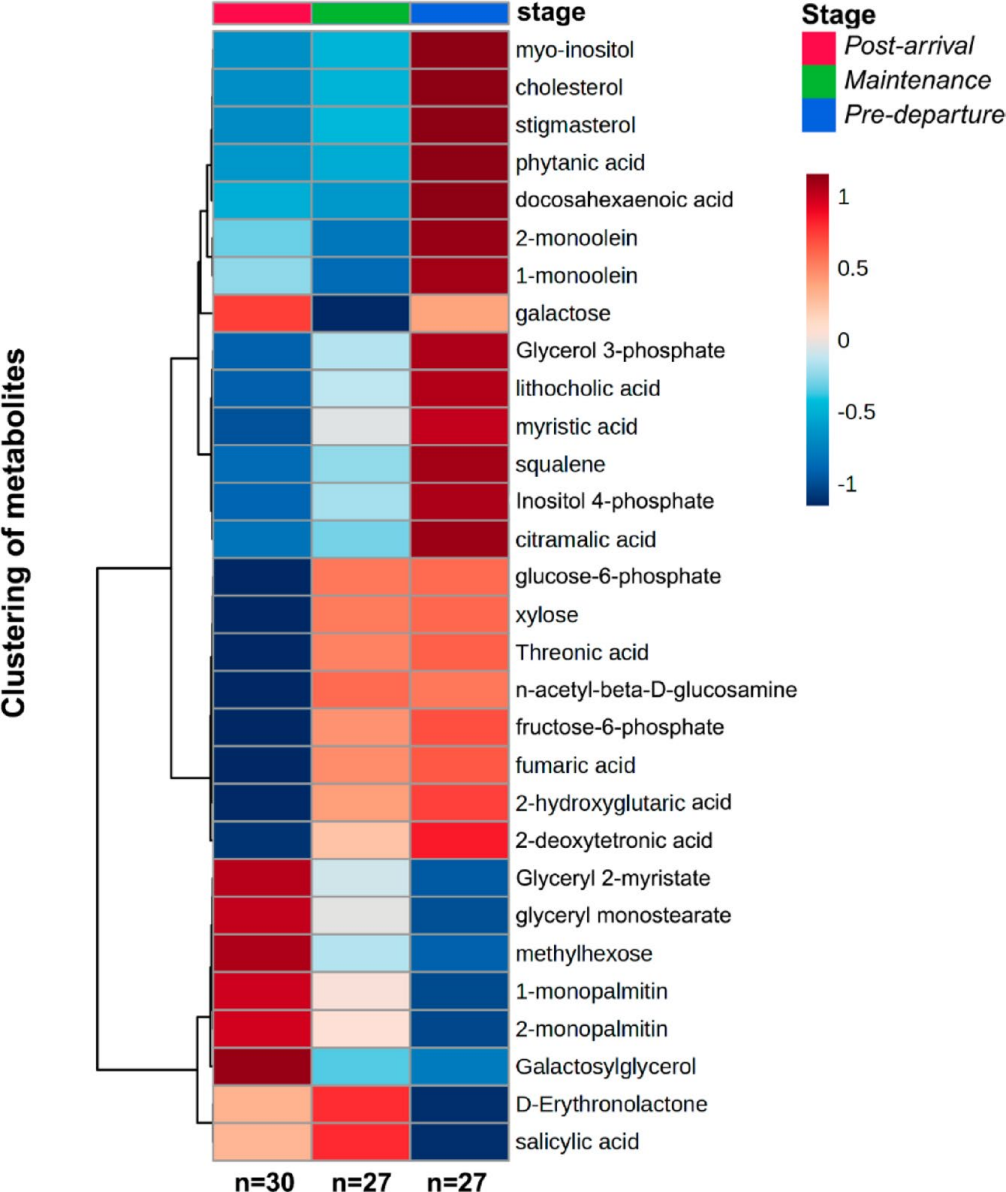
Hierarchical clustering heatmap for top 30 plasma metabolites in Hudsonian godwits (*Limosa haemastica*), selected based on one-way ANOVA p-values across non-breeding stages. Metabolite values represent stage-averaged normalized intensities for stages. The sample size for each stage is indicated below each column.

The 30 metabolites with the lowest ANOVA-adjusted p-values were used to assess temporal changes in godwit metabolism. A stage-averaged hierarchical clustering heatmap (Fig. 4) revealed broad metabolic differences among stages, with pre-departure showing the most distinct profile relative to earlier stages. Consistent with the PLS-DA, samples grouped by stage along a continuum from *post-arrival* to *pre-departure*. Individuals from the *maintenance* stage clustered with both *post-arrival* and *pre-departure* individuals in the heatmap, reinforcing the interpretation of *maintenance* as a transitional metabolic state (Supp. Figure 3). The clustering identified three major groups of metabolites that were predominantly upregulated during the *pre-departure* stage, compared with earlier stages, representing a metabolomic signature of the fattening process in preparation for northbound migration. These metabolites were primarily associated with lipid metabolism, including glycerolipid metabolism (e.g., 1- and 2- monoolein, glycerol 3-phosphate), bile acid and/or steroids biosynthesis (e.g., cholesterol, lithocholic acid, stigmasterol, squalene), biosynthesis of saturated and unsaturated fatty acids (e.g., phytanic acid, docosahexaenoic acid, 2-deoxytetronic acid, myristic acid). Additional pathways enriched at this stage included butanoate metabolism (e.g., 2-hydroxyglutaric acid), glycan metabolism, such as glycosaminoglycan biosynthesis (e.g., xylose), and carbohydrate metabolism, notably galactose (e.g., galactose, myo-inositol, inositol 4-phosphate), and starch and sucrose metabolism (e.g., glucose-6-phosphate, fructose-6-phosphate). In contrast, a smaller cluster of metabolites was significantly upregulated in individuals from the *post-arrival* stage and, to a lesser extent, in some *maintenance*-stage individuals. These compounds were mainly associated with glycerolipid metabolism, including 1 - and 2- monopalmitin, galactosylglycerol, glyceryl 2-myristate, and glyceryl monostearate, highlighting a signature of lipid catabolism and recovery immediately following migration.

### *Post-arrival* and *pre-departure* phenotypes comparison

**Fig. 5:**
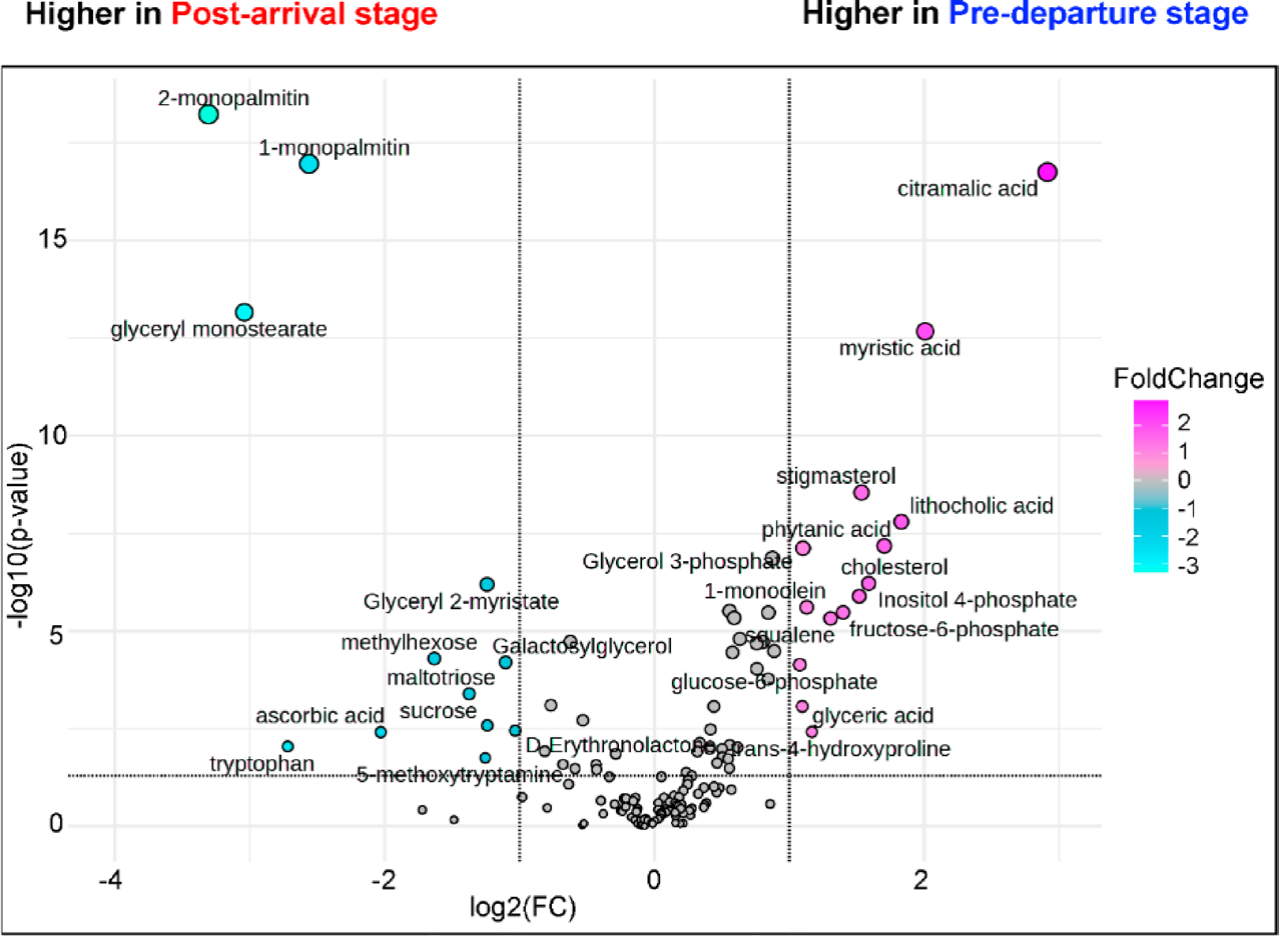
Pairwise comparison between feature intensities from the *post-arrival* and the *pre-departure* stage. x-axis: log₂ fold change (FC) calculated as the ratio *pre-departure/post-arrival* of normalized feature intensity. Negative values indicate features with higher intensity in the *post-arrival* stage, whereas positive values indicate features with higher intensity in the *pre-departure stage*. y-axis: −log₁₀(p) from pairwise t-test. Significance threshold was set to *p* < 0.05 for pairwise subgroup comparisons.

Twelve metabolites were significantly more abundant in the *post-arrival* stage, while 14 metabolites were enriched in individuals preparing for migration at the *pre-departure* stage. *Post-arrival* profiles were dominated by glycerolipid metabolism, particularly driven by elevated levels of 1- and 2- monopalmitin and glyceryl monostearate. In contrast, *pre-departure* profiles reflected pathways linked to energy storage and mobilization, characterized by metabolites associated to the biosynthesis with the saturated and unsaturated fatty acids (e.g., myristic acid), bile acid and steroids biosynthesis (e.g., cholesterol, lithocholic acid, and stigmasterol), glycolysis/gluconeogenesis (e.g., glucose-6-phosphate and fructose-6-phosphate), and fructose and mannose metabolism (e.g., phytanic acid) (Fig. 5).

**Fig. 6:**
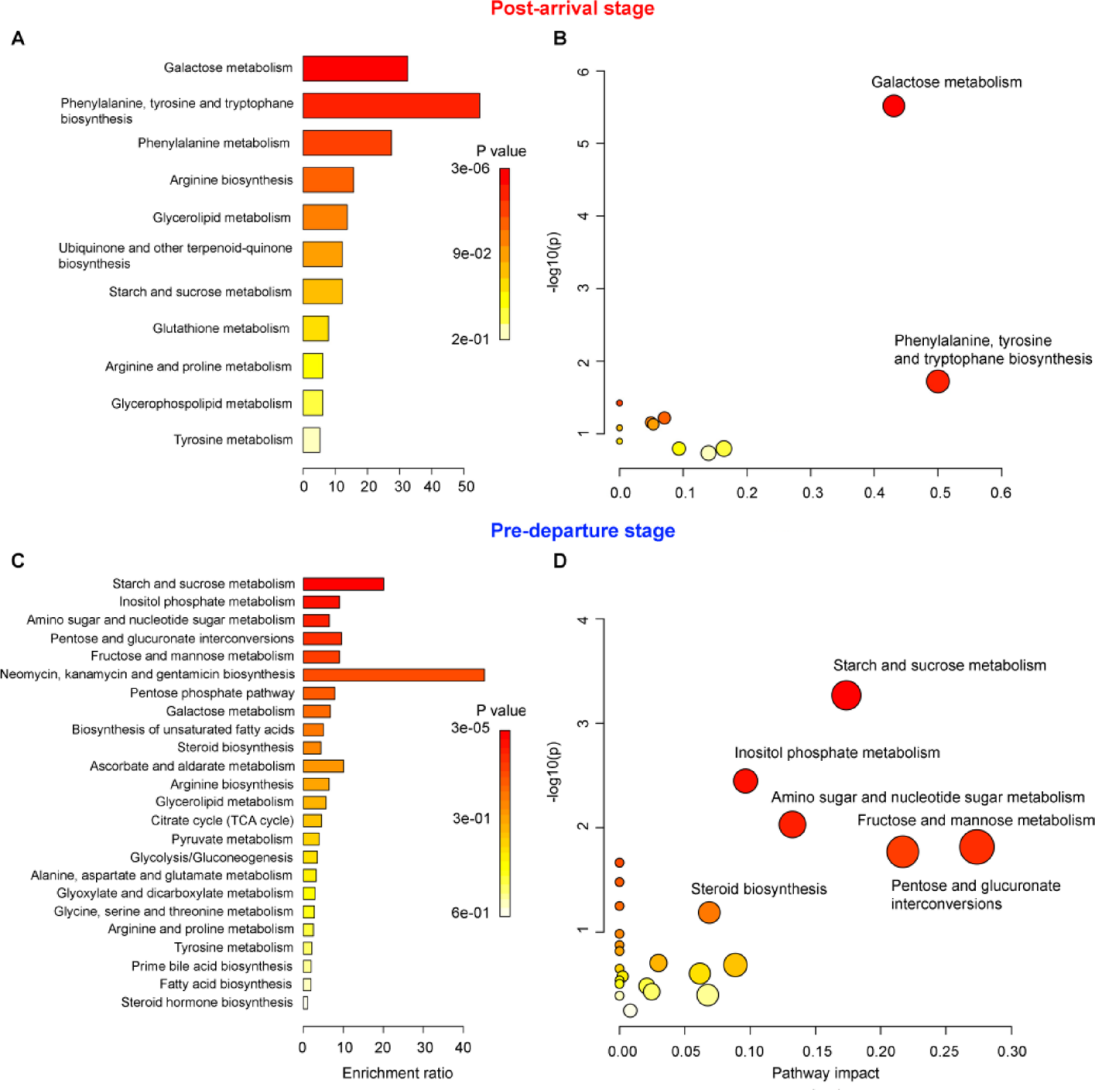
**(A**,** C)** Significative annotated metabolite list of *post-arrival* and *pre-departure* stage submitted to MetaboAnalyst for metabolite class enrichment analysis, compared against KEGG pathways implemented in MetaboAnalyst for metabolite class enrichment analysis. **(B**,** D)** Pathway impact and statistical significance of the metabolic pathways identified through pathway enrichment analysis of *post-arrival* and *pre-departure* stage. Higher impact values represent the relative importance of the pathway; the size of the circle indicates the impact of the pathway while the color represents the significance (the more intense the red color, the lower the p).

To further explore these differences, the 49 metabolites that varied significantly among stages were subjected to pathway and enrichment analysis (Fig. 6). In the *post-arrival* stage, the five most altered metabolite sets included galactose metabolism, phenylalanine/tyrosine/tryptophane biosynthesis, phenylalanine metabolism, arginine biosynthesis, and glycerolipid metabolism (Fig. 6A-B). In contrast, for the *pre-departure* stage, enrichment analysis highlighted starch and sucrose metabolism, inositol and phosphate metabolism, amino sugar and nucleotide sugar metabolism, pentose and glucoronate interconversions, and fructose and mannose metabolism as the most altered pathways (Fig. 6C-D). Pathway impact analysis confirmed that galactose metabolism and aromatic amino acid biosynthesis were the most strongly impacted pathways in the *post-arrival* stage (Fig. 6B), whereas carbohydrate remodeling and sugar metabolism pathways dominated during *pre-departure* stage (Fig. 6D).

## Discussion

In this study, we uncovered progressive shifts in metabolite abundance across three distinct physiological stages related to endurance migration, *post-arrival*, *maintenance*, and *pre-departure.* These results highlight the remarkable phenotypic flexibility of long-distance migratory shorebirds, demonstrating how their metabolic profiles dynamically adjust to meet contrasting seasonal demands, from the recovery of prolonged migratory flight, followed by a stabilization period, and the physiological preparation for the subsequent long-distance flight to breeding grounds. Our findings underscore that migration is supported not by static metabolic states but by a dynamic strategy of flexible, stage-specific metabolic reprogramming that allows birds to efficiently allocate metabolic resources across the annual cycle.

### Seasonal metabolic transition

Metabolomic profiles showed a clear temporal trajectory, with *post-arrival* and *pre-departure* stages showing distinct separation and the *maintenance* stage occupied an intermediate transitional phenotype. This pattern, supported by both PLS-DA analysis and hierarchical clustering, indicates that the migratory phenotype is not discrete but instead represents a physiological continuum that gradually changes across the non-breeding season. Such transitions are likely shaped by the interplay between environmental and behavioral signals synchronized by endogenous circannual rhythms^11,12^. Importantly, this transition culminates in the *pre-departure* stage characterized by a distinct metabolomic signature that reflects extensive metabolic reprogramming associated with fattening. This upregulation of key pathways suggests that pre-migratory fattening is not merely a passive reserve accumulation but rather a coordinated, systemic adjustment that optimizes energy stores and physiological resilience for endurance migrations (e.g. trans-hemispheric or trans-oceanic flights). These extreme seasonal transitions are comparable to those taxa that its physiology is strongly modulated by circannual rhythms. For example, American black bears (*Ursus americanus*) have metabolic shifts that conserve energy and preserve muscle during hibernation^34^, while moor frog (*Rana arvalis*) accumulate cryoprotectants in muscle and liver tissues to tolerate internal freezing^35^⁠. These parallels highlight the broader principle of metabolic flexibility as an adaptive response to extreme seasonal challenges, whether migration, hibernation, or freeze tolerance, by finely tuned metabolic transitions, synchronized by endogenous circannual rhythms and environmental signals.

### Metabolomic profile of the Post-arrival stage

The *post-arrival* metabolomic profile reflects the initial physiological transition as godwits recover from their long migratory flight. This stage was characterized by metabolic signatures of muscular regeneration, energy rebalancing, lipid catabolism, and antioxidant activity. Together, these patterns suggest coordinated metabolic adjustments required to restore homeostasis after endurance migration.

After a long-distance migratory flight, birds experience significant muscle catabolism and depletion of energy reserves, requiring rapid physiological recovery^12^. Particularly, an elevated concentrations of monoacylglycerols (1- and 2- monopalmitin, glyceryl monostearate, and glyceryl 2-myristate), the most upregulated metabolites during this stage (see Fig. 5), suggest an active turnover of residual fat reserves remaining after migratory flight^36^⁠. These compounds represent intermediate products of triacylglycerol hydrolysis indicative with ongoing lipolysis^37^, releasing fatty acids and glycerol to sustain basal energy production during the recovery phase. In addition to supporting immediate energetic requirements, lipid catabolism may contribute to membrane remodeling and cellular repair processes^38^ following prolonged endurance flight during which membranes and organelles are expected to experience substantial mechanical and oxidative stress. Thus, the *post-arrival* stage represents a transitional metabolic state in which remaining lipid reserves are strategically mobilized to facilitate physiological recovery.

The upregulation of galactose metabolism, aromatic amino acid (e.g., phenylalanine, tyrosine, and tryptophan) biosynthesis, and arginine biosynthesis suggests activation of pathways essential for muscle repair and functional recovery. Galactose, converted into glucose derivatives, supports muscle glycogen replenishment^39^ and contributes glycoproteins synthesis, which supports muscle cell repair and structural integrity^40^ and facilitates protein trafficking and membrane organization within muscle cells^41^. In addition, by enhancing mitochondrial function and metabolic flexibility, galactose metabolism enables skeletal muscle cells to switch efficiently between energy substrates^42^⁠. Together, these mechanisms suggest that an upregulation of galactose metabolism is crucial for muscular repair and functional recovery following migration.

Similarly, aromatic amino acids play a pivotal role in regulating muscle regeneration and protein synthesis^43,44^. Phenylalanine, tyrosine, and tryptophan, obtained through diet or gut microbiota^45^⁠, optimize nutrient utilization improving feed efficiency and growth performance^46^, activate anabolic pathways promoting muscle protein synthesis^47^ and stimulates myogenic gene expression (e.g., *MyoD* and *myogenin*), enhancing muscle growth and repair^43^.

Arginine is especially important in uricotelic species, contributing to growth, energy metabolism, immune response, and tissue repair^48,49^. Through conversion to ornithine, proline, and hydroxyproline, arginine supports tissue repair collagen turnover^50^⁠, critical for rebuilding muscle and connective tissue. Arginine also serves as a precursor for nitric oxide, facilitating vasodilation and improving oxygen and nutrient delivery to damaged muscle tissues^51,52^⁠ and for creatine synthesis, rapidly replenish ATP during intense muscle activity^53,54^⁠. Elevated creatine kinase levels observed in migratory shorebirds following long flights, further indicates reliance on creatine metabolism during recovery after muscular stress^55^⁠.

Increased levels of antioxidants, including ascorbic acid, tryptophan, 5-Methoxytryptamine, and trans-4-hydroxyproline^50,56–58^⁠, are consistent with responses to oxidative stress incurred during sustained flight^12,59^. High-intensity exercise generates reactive oxygen species that can damage lipids, proteins, and DNA^60^. Migratory birds rely on flexible antioxidant defense systems that are dynamically regulated across the migratory cycle^10,22,61,62^. Hence elevated antioxidant metabolites in *post-arrival* godwits demonstrate activation of protective pathways to mitigate oxidative damage and restore physiological balance after migration.

### Metabolomic profile of the Maintenance stage

The metabolomic profile during the *maintenance* stage reflects a period of physiological stability, in which birds are no longer challenged with acute demands of post-arrival recovery nor yet engaged in the intense metabolic reprogramming of pre-departure fattening. Instead, birds in this stage appear to prioritize energy conservation, metabolic stability, and gradual preparation for the next migratory cycle.

Metabolites that peaked during the maintenance stage were predominantly simple carbohydrates (e.g. 1,5-anhydroglucitol, xylulose, and fructose), indicative of a metabolic state characterized by baseline carbohydrate turnover and limited anabolic or catabolic pressure. This profile suggests that birds maintain essential energetic functions without engaging in accelerated energy storage or tissue remodeling, consistent with a low-demand physiological state^63^. Supporting this pattern, galactose showed its lowest average abundance during this stage. Galactose is a key monosaccharide involved in both energy production and the biosynthesis of complex molecules; however, its entry into central metabolism is tightly regulated and typically increases under conditions of high energetic demand or tissue repair^64^. Metabolic pathways associated with tissue repair and oxidative stress, which dominate during the *post-arrival* profile, were progressively downregulated, whereas early shifts toward lipid accumulation began to emerge through the gradual upregulation of lipid-related pathways linked to fat deposition. These patterns suggest that the *maintenance* stage functions as a metabolic bridge, balancing recovery and preparation and ensuring later redirection of resources efficiently into rapid fattening required for subsequent long-distance flight.

### Metabolomic profile of the Pre-departure stage

The *pre-departure* metabolomic profile reflects the extensive physiological reprogramming undertaken by birds to prepare for their longest endurance flight within the annual cycle. This stage was characterized by pronounced shifts in carbohydrate and lipid metabolism, including upregulation of sugar-processing metabolism, inositol phosphate metabolism, and the biosynthesis of steroid and unsaturated fatty acids. These changes suggest a coordinated strategy of active energy reserves remodeling, endocrine modulation, and enhanced biosynthetic capacity to generate efficient fuel reserves capable of sustaining endurance migration.

Several carbohydrate-related pathways were significantly upregulated during the pre-departure stage, indicating an active reorganization of central carbon metabolism in preparation for migration. The mobilization of complex sugars and phosphorylated intermediates, including glucose-6-phosphate, fructose-6-phosphate, myo-inositol, inositol 4-phosphate, galactose and xylose, supports enhanced glucose availability through pathways such as sucrose, fructose, and mannose metabolism. Increased glucose production likely contributes to glycogen accumulation, providing a rapidly mobilizable energy source useful during sustained muscular activity^65^. In parallel, glucose-6-phosphate can be diverted into the pentose phosphate pathway, generating NADPH, a cofactor required for fatty acid biosynthesis and for maintaining redox balance^66,67^⁠. This functional connection between glucose metabolism, lipid synthesis, and antioxidant defense underlines the importance of sugar pathways in fueling not only immediate energy demands but also supporting anabolic processes critical to flight endurance performance^68^⁠. Consistent with findings in migratory passerines^69^ and shorebirds^70^, these pathways are suggested to be essential for pre-migratory *de novo* lipogenesis, converting glucose into fatty acids and rapidly replenishing fat stores. Thus, godwits are likely to employ a dual metabolic priming strategy, mobilizing carbohydrates both to supply readily accessible glycogen and to provide substrates for lipid synthesis. Within this broader metabolic shift, citramalic acid emerged as the most enriched metabolite during this stage (Fig. 5). In other biological systems, citramalic acid has been associated with central carbon metabolism, at the interface between carbohydrate processing and lipid biosynthesis^71^ and its accumulation has been interpreted as a marker of metabolic reprograming favoring lipogenesis once immediate energy demands are met. Although its specific function in avian metabolism remains unclear, the elevated levels observed in godwits are consistent with a reorganization of central carbon metabolism that supports energy storage and lipid synthesis during pre-migratory fattening.

Lipid metabolism was strongly remodeled, as evidenced by pronounced shifts in fatty acid composition and lipid-related pathways (Fig. 5). These changes included increased abundances of saturated fatty acids (e.g., myristic acid), together with mono- and polyunsaturated fatty acids (e.g., oleic acid and docosahexaenoic acid), as well as sterols and sterol-related metabolites (e.g., cholesterol, stigmasterol, squalene, and lithocholic acid). Myristic acid was the most significant upregulated saturated fatty acids, representing compact, energy-dense lipid reserves that are well suited for long-term fuel storage prior to departure, providing a stable energy pool for prolonged migratory flights^12^. In contrast, polyunsaturated fatty acids are preferentially mobilized and oxidized during endurance exercise, enhance mitochondrial efficiency, and optimize membrane fluidity under high metabolic rates, thereby promoting effective fatty acid utilization in flight muscles and supporting sustained aerobic performance^12,72^. Although our metabolomic approach did not directly assess isotopologue distributions or deuterium enrichment patterns, recent work has proposed that carbohydrate remodeling toward unsaturated fatty acid biosynthesis may contribute to mitochondrial protection during high-energy-demand states through selective deuterium depletion within metabolic pathways^73,74^. In this context, the observed enrichment of sugar metabolism prior to departure may serve not only as an energy supply mechanism but also as a preparatory step for optimizing mitochondrial redox efficiency and preserving nanomotor ATPase function under sustained migratory exertion.

Elevated levels of sterols and sterol-related metabolites further indicate hormonal and metabolic modulation associated with the physiological transitions required for migration. Cholesterol, as a precursor of steroid hormones such as corticosterone, supports hyperphagia, promotes fat deposition, and enhances muscle hypertrophy^75–77^⁠, processes aligned with the metabolic priorities of *pre-departure* stage. Metabolites involved in bile acid biosynthesis, including cholesterol and squalene, were also elevated. Although pathway-level enrichment was not statistically significant, this pattern suggests increased bile acid activity, crucial for lipid emulsification and absorption^78^. Enhanced bile acid metabolism during hyperphagia ensures efficient assimilation of dietary lipids and supports gut health^79^⁠, thereby facilitating the rapid accumulation of fat reserves required to sustain prolonged endurance flights in migratory birds.

Our study demonstrates that *Limosa haemastica* exhibits significant metabolomic flexibility across the non-breeding season, reflecting the progressive transitions of distinct physiological phenotypes associated with the energetic demands during the annual cycle. Birds exhibit signatures of muscle repair, lipid catabolism, and antioxidant defense reflecting the immediate recovery from long-distance flight. After that, birds show a stabilized (intermediated) phenotype, marked by downregulation of tissue repair pathways and gradual upregulation of lipid storage, representing a bridge between recovery and fueling. Finally, birds display a metabolomic reprogramming characterized by activation of carbohydrate remodeling, unsaturated fatty acids synthesis, and steroid metabolism, supporting rapid fattening and endocrine modulation prior to trans-hemispheric migration. The progressive transition among these stages highlights the extraordinary phenotypic flexibility of migratory shorebirds, revealing how flexible metabolic strategies enable animals to cope with some of the most demanding physiological challenges in nature.

## Methods

### Study area and sample collection

We sampled individuals from a population of Hudsonian godwits that spent the austral summer on different bays on Chiloé Island, Southern Chile^80,81^. These birds arrived on average from early October up to early November and remain there for approximately six months throughout the non-breeding season^32^. In March, they continue to accumulate fat reserves, a process that begins in mid-February, in preparation for northward migration to their breeding grounds, which occur around the second week of April^82^.

A total of 84 adult godwits were captured at high tide using cannon nets in Pullao Bay (42°28 S, 73°41 W) during the austral summer of the 2021–2022 season. The captures were divided into three sampling stages to assess the temporal dynamics of physiological and behavioral changes associated with the phenotypic stages of the non-breeding season. By November 15th, 30 individuals were sampled (23 males, 7 females), approximately two to six weeks after their arrival at their non-breeding grounds, referred as the *post-arrival* stage. In January 22nd, we sampled 27 individuals (19 males, 8 females) associated to an intermediate period, referred as *maintenance* stage. Finally, in March 30th, we sampled 28 individuals (14 males, 14 females) representing the last days of the fattening period prior to the departure for a long-distance migratory flight to the breeding grounds, referred as the *pre-departure* stage.

Once captured, birds were maintained in individual cages for no longer than 2 h before processing. Each individual was weighed, ringed, and aged according to the molting stage^83^, always by the same person (JGN). Standard morphological measurements of the tarsus were then taken to calculate the scale mass index (SMI)^84^, which was used as a proxy for body condition. This index was performed separately for female and male, as there is strong inverse sexual size dimorphism (~ 25% size difference)^85^⁠. The fat score was estimated following the classification scheme for scoring subcutaneous fat deposits in shorebirds^86^⁠. Sex was determined based on bill length, following the criterion established by Gherardi-Fuentes et al. (2020). Molecular sexing was conducted for individuals whose sex could not be assigned morphologically^87^.

Blood samples (1 mL) were obtained from the right jugular vein using a sterile syringe, deposited in a heparin tube (1,5 mL), and kept on ice for no more than 4 h until centrifugation at 10,000 g for 10 min. The obtained plasma was transferred to a cryogenic tube (2 mL) and immediately frozen in liquid nitrogen. The samples were then stored at −80 °C until further analysis. After sampling, birds were released immediately at the capture site.

### Sample preparation

Each plasma sample was cleaned to remove impurities and incompatible molecules, ensuring robust and reliable results. A 100 µL aliquot of plasma was added to 1 mL of acetonitrile/isopropanol/water (3:3:2) containing 1 µL of ribitol (4 mg/ml, Sigma-Aldrich), used as an internal control metabolite. Samples were vortexed, centrifuged, and 450 µL of supernatant was evaporated to dryness using a SpeedVac concentrator (Savant^®^ SPD131DDA, Thermo Fisher Scientific, USA), reconstituted with 450 µL of acetonitrile/water (1:1) and repeating the evaporation process. Then, 2 µL of a retention rate marker (FAME mixture, 400505, Fiehn GC/MS Metabolomics Standards Kit, Agilent, CA, USA) were added, followed by 10 µL of methoxyamine hydrochloride (MeOX)/pyridine hydrochloride (20 mg/mL, Sigma-Aldrich) and incubated at 30 °C for 90 min. Afterward, 90 µL of MSTFA (N-methyl-N-trifluoroacetamide) with 1% TMCS (trimethylchlorosilane) derivatizing agent (Thermo Fisher Scientific, Pierce Biotechnology, Rockford, IL) was added and incubated at 37 °C for 30 min. Samples were then transferred to a glass vial insert (250 µL, Agilent, Santa Clara, CA) for GC/MS analysis.

### Metabolomics by GC/MS

Metabolites in the plasma samples were separated by gas chromatography (GC) and quantified by mass spectrometry (MS). Samples were injected into an Agilent 7890B GC system coupled to a 5977 A Mass Selective Detector (Agilent Technologies, Palo Alto, CA, USA) in electron impact ionization mode, using an Agilent 7693 Series Autosampler. Derivatized samples (1 µL) were injected in splitless mode onto a 30 m × 0.25 mm × 0.25 μm DB-5 column (Agilent Technologies). The injector temperature was set at 250 °C, with a carrier gas flow rate of 1 mL/min. The oven temperature started at 60 °C and was increased at a rate of 10 °C/min until it reached 325 °C, with a total run time of 37.5 min. All derivatized samples were analyzed within 24 h of preparation. A FAME standard solution was injected to determine retention times and calculate metabolite retention indices.

### Identification of metabolites

Metabolite identification followed the method described by Kind et al. (2009)^88^⁠. Briefly, peak detection, deconvolution, and peak alignment were performed using MSDIAL 2.83 (RIKEN Center for Sustainable Resource Science, Yokohama, Japan) to process the total ion chromatogram and electron ionization-MS (EI-MS) spectra. Mass spectra of the trimethylsilylated metabolites were matched against mass spectral libraries in NIST MSP format. Matches were ranked based on retention index and spectral similarity, using the Fiehn retention index based on FAME. Metabolites were identified by comparing their EI-MS spectra with reference compounds from the NIST or Fiehn libraries. Identification criteria included a retention index tolerance of 2,000, an EI similarity cutoff of 65%, a score cutoff of 70%, a mass scale tolerance of 0.5 Da, and a retention time tolerance of 0.5 min.

### Statistical analysis

To evaluate seasonal differences in body condition, we used one-way ANOVAs to test for changes in scaled mass index (SMI) across non-breeding stages, separately for females and males. SMI was log-transformed in males to meet normality assumptions. Post hoc pairwise comparisons were conducted using Tukey’s HSD method. These analyses were performed in R using the *stats*^89^ and *emmeans*^90^ packages.

Metabolomic data were statistically analyzed using MetaboAnalyst v6.0 (Xia Lab, McGill University, Canada; http://www.metaboanalyst.ca)^91^ and R version 4.05^89^, following previously published protocols^85^⁠. Metabolic pathway enrichment and pathway topology analyses were conducted using KEGG pathway database^92,93^ implemented in MetaboAnalyst. Metabolites with more than 50% missing values or values below the detection limit were excluded from the analysis. Concentrations were normalized using ribitol as an internal standard, following logarithmic transformation and mean scaling to achieve a Gaussian distribution. Metabolite names and chemical synonyms of annotated features were identified through the Human Metabolome Database (https://hmdb.ca/), PubChem (https://pubchem.ncbi.nlm.nih.gov/), and KEGG (Kyoto Encyclopedia of Genes and Genomes, https://www.genome.jp/kegg/).

Partial least squares-discriminating analysis (PLS-DA) and variable importance in projection (VIP) scores were calculated. Cross-validation and permutation tests were used to estimate the PLS-DA model, with a sum of squares captured by the model R2 > 0.87 and p-value = 0.0005 (0/2000), respectively. Permutational multivariate analysis of variance (perMANOVA) was performed using Bray-Curtis dissimilarity to assess differences on the metabolomics profiles between stages as a main factor and sex as covariate, using the adonis function in the *vegan* package^94^⁠. Hierarchical cluster analysis was performed using Euclidean distance and Ward`s clustering algorithm to generate the heatmaps based on stage-averaged samples and individual samples. A dendrogram based on the normalized concentration of the top 30 metabolites was assessed by ANOVA or t-test p-value for clustering each individual sample in the stage groups. Enrichment analysis was conducted using a unified list of annotated compounds and compared against RefMet chemical super class (Metabolomics Workbench, https://www.metabolomicsworkbench.org) and KEGG metabolite pathways (https://www.genome.jp/kegg/*).* The *Taeniopygia guttata* (zebra finch) library was used as the reference, and a hypergeometric test was applied for overrepresentation analysis. Subgroup analysis for pairwise volcano plots was conducted separately for comparison between *post-arrival* and *pre-departure* stages with a t-test threshold of *p* < 0.05 after FDR correction. Pathway topology analysis was performed separately for each stage by selecting only metabolites that showed significant differences.

### Approval for animal experiments

This study was conducted in accordance with the guidelines granted by the Agricultural and Livestock Service (SAG, Resolution No. 5932/2016 and 7625/2018) and approved by Universidad Austral de Chile’s Institutional Animal Care and Use Committee (CICUA) (Protocol No. 355/2019). The study is reported in accordance with ARRIVE guidelines.

## Supplementary Information

Below is the link to the electronic supplementary material.

Supplementary Material 1

## Data Availability

The datasets generated and analyzed during the current study are available from the corresponding authors upon reasonable request.
