# Supplementary material for: Metabolomic profiles reveal physiological transitions required for long-distance avian migration
Source: Sci Rep. 2026 Apr 4;16:16344. doi: 10.1038/s41598-026-41603-2 (PMC13212997; doi:10.1038/s41598-026-41603-2)
Supplement: Supplementary file 1 — Supplementary Material 1 [file 41598_2026_41603_MOESM1_ESM.pdf]

Supplementary information

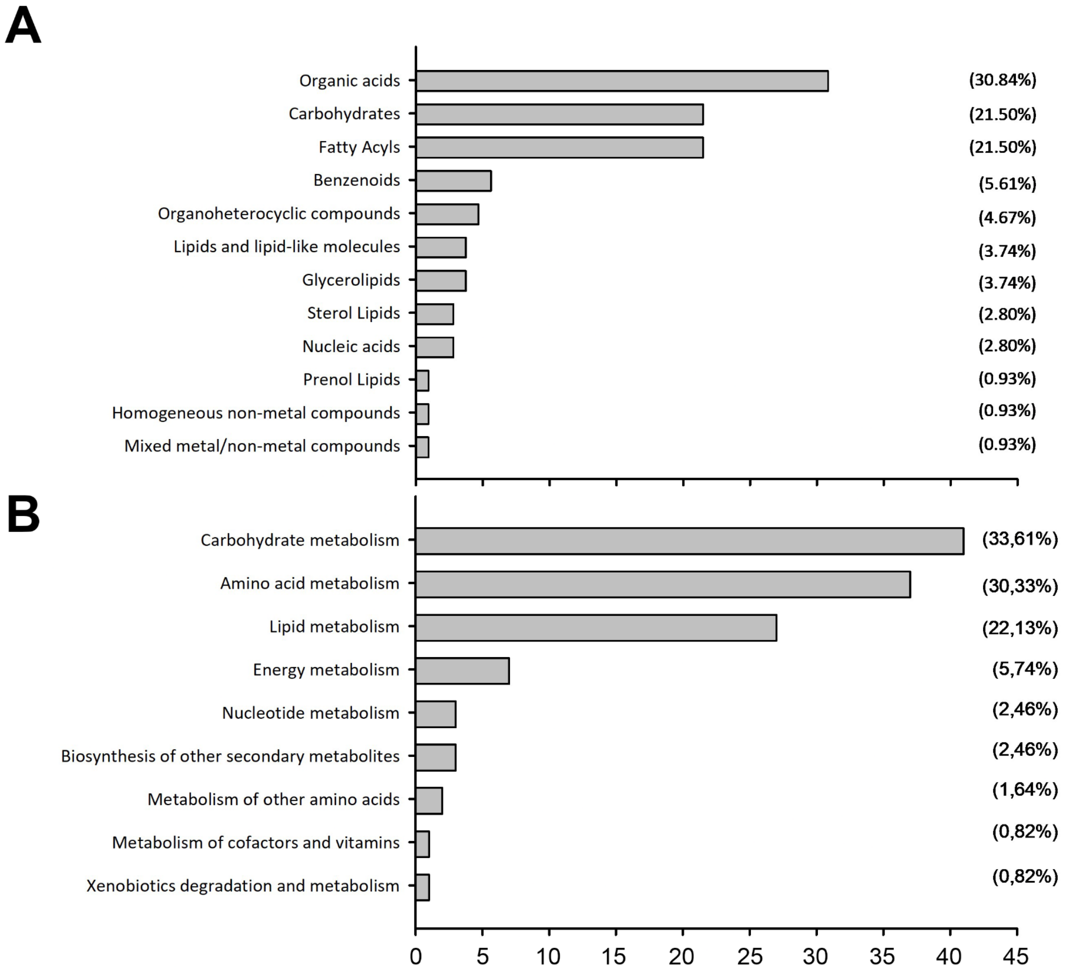

Supplementary Figure 1. Metabolomic profile of plasma in the different periods of non breeding season. A. Classification of 107 metabolites detected by gas chromatography-mass spectrometry (GC-MS) in plasma according to the chemical class in PubChem. Each bar represents the relative abundance of the chemical class. The absolute and relative abundance (%) of each class is also indicated. B. in KEGG

Supplementary Figure 2. Violin plots of the 49 metabolites with significant differences among non-breeding stages of godwits. Each plot shows the distribution of concentrations across individuals (density, median, interquartile range). Metabolites are ordered alphabetically and labeled on the x-axis. For each metabolite, both the original concentration (Original conc.) and the log-transformed normalized concentration (Normalized conc.) used for statistical analyses (ANOVA with Fisher's LSD) are presented. Stages are labeled as I. early (*post-arrival*), II. mid (*maintenance*), and III. late (*pre-departure*).

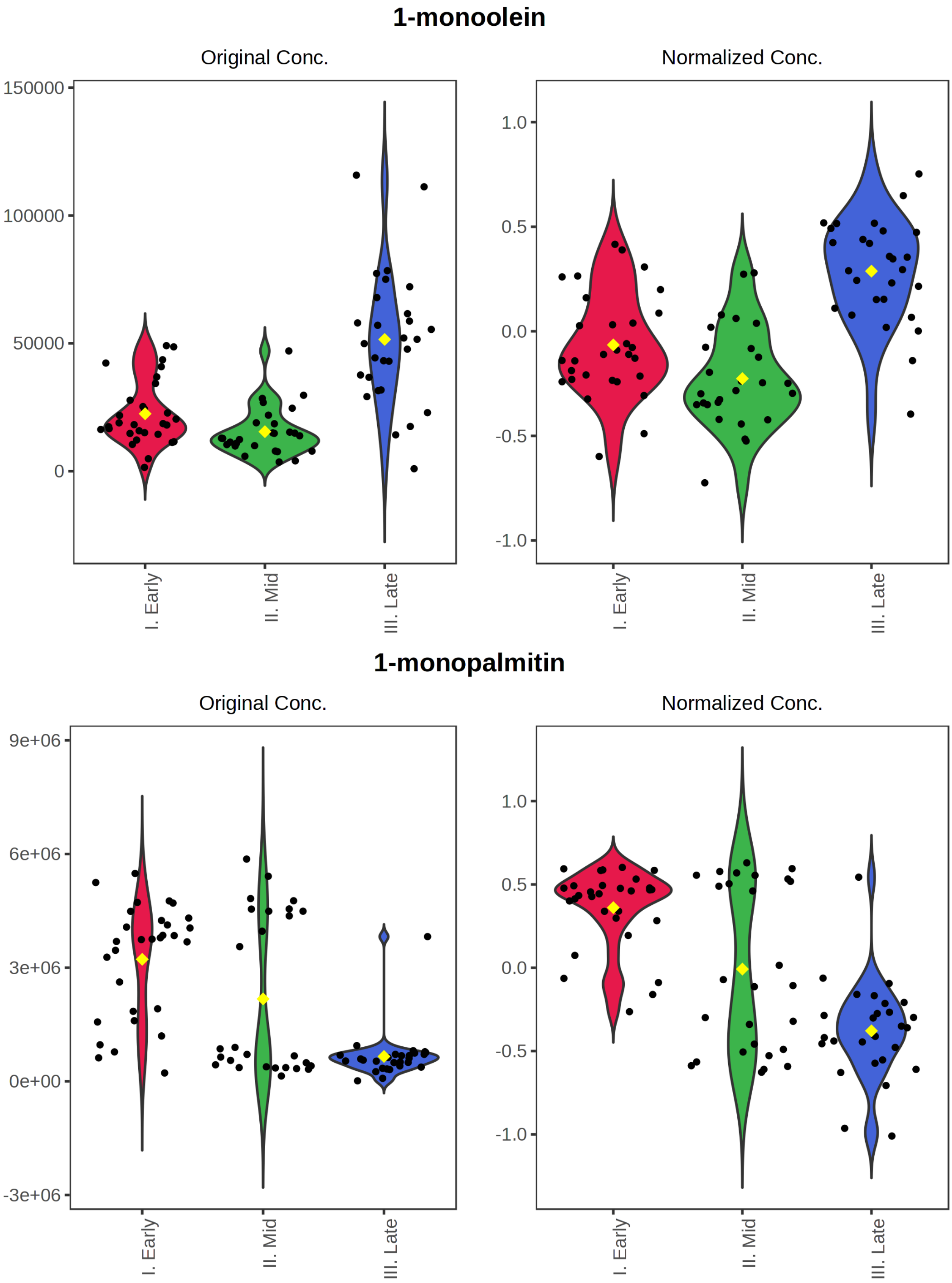
1-monoolein
Original Conc.
Normalized Conc.
150000
100000
50000
0
1.0
0.5
0.0
-0.5
-1.0
I. Early
II. Mid
III. Late
1-monopalmitin
Original Conc.
Normalized Conc.
9e+06
6e+06
3e+06
0e+00
-3e+06
1.0
0.5
0.0
-0.5
-1.0
I. Early
II. Mid
III. Late

2-deoxytetronic acid

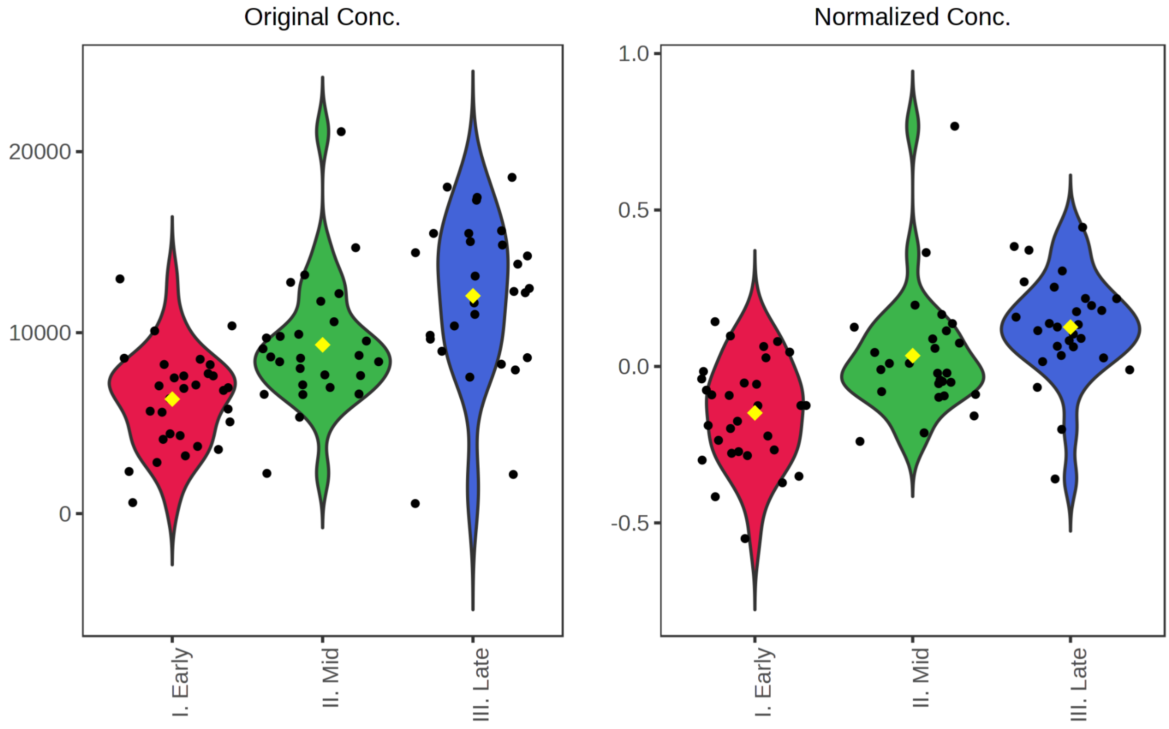

2-hydroxyglutaric acid

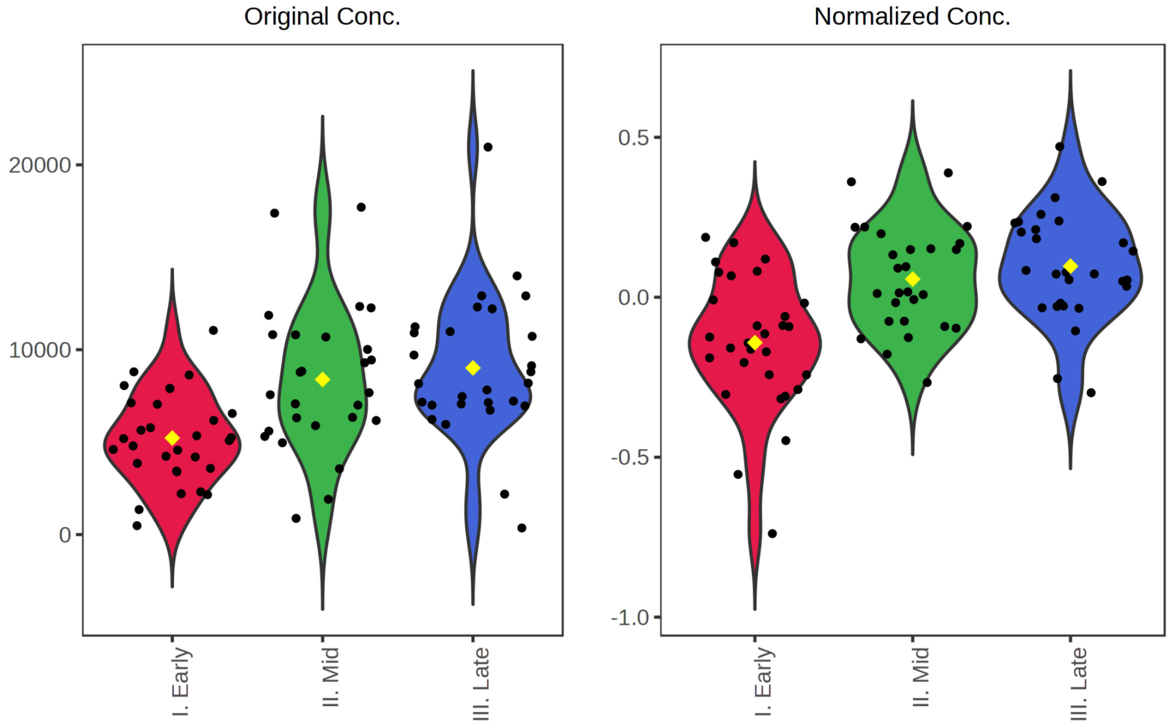

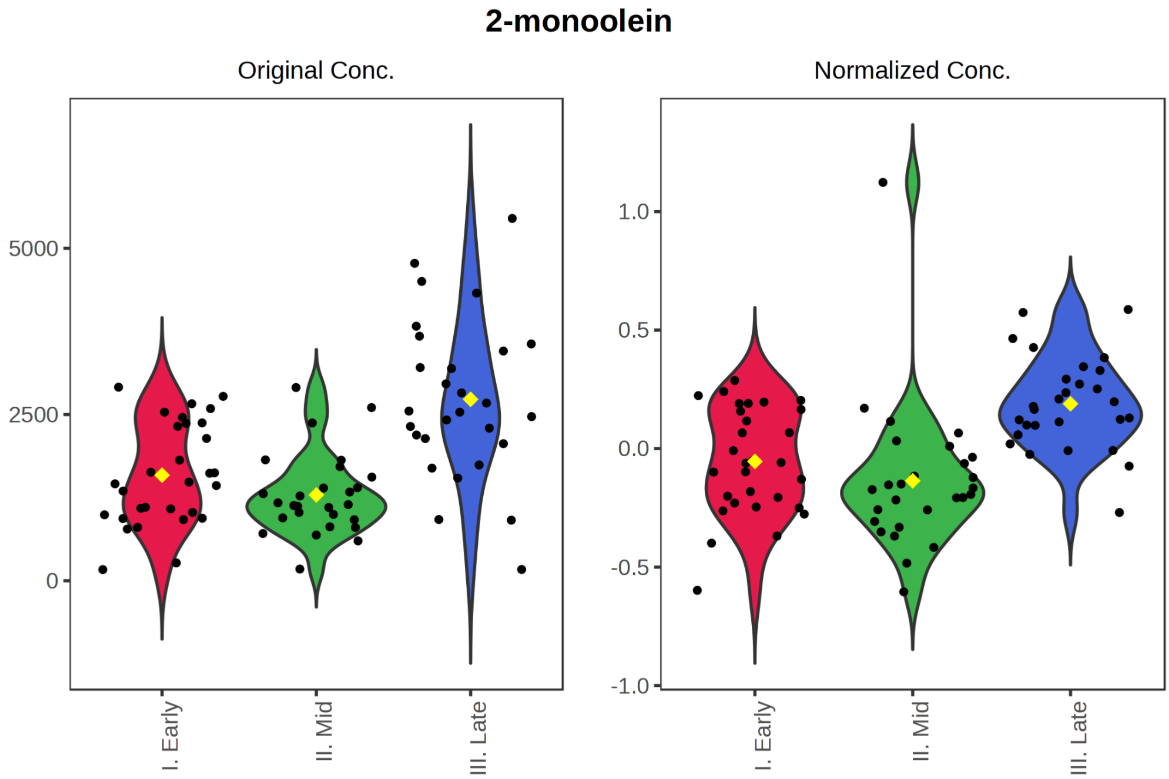
2-monoolein
Original Conc.
Normalized Conc.
5000
2500
0
1.0
0.5
0.0
-0.5
-1.0
I. Early
II. Mid
III. Late

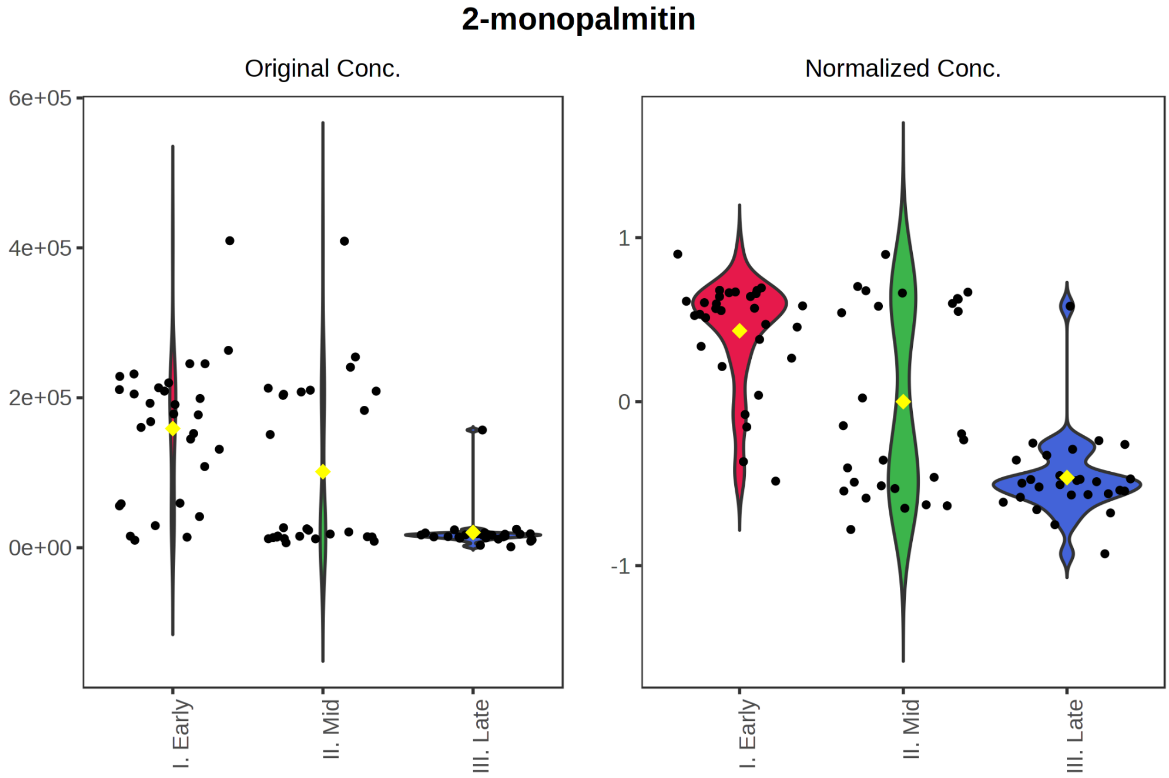
2-monopalmitin
Original Conc.
Normalized Conc.
6e+05
4e+05
2e+05
0e+00
1
0
-1
I. Early
II. Mid
III. Late

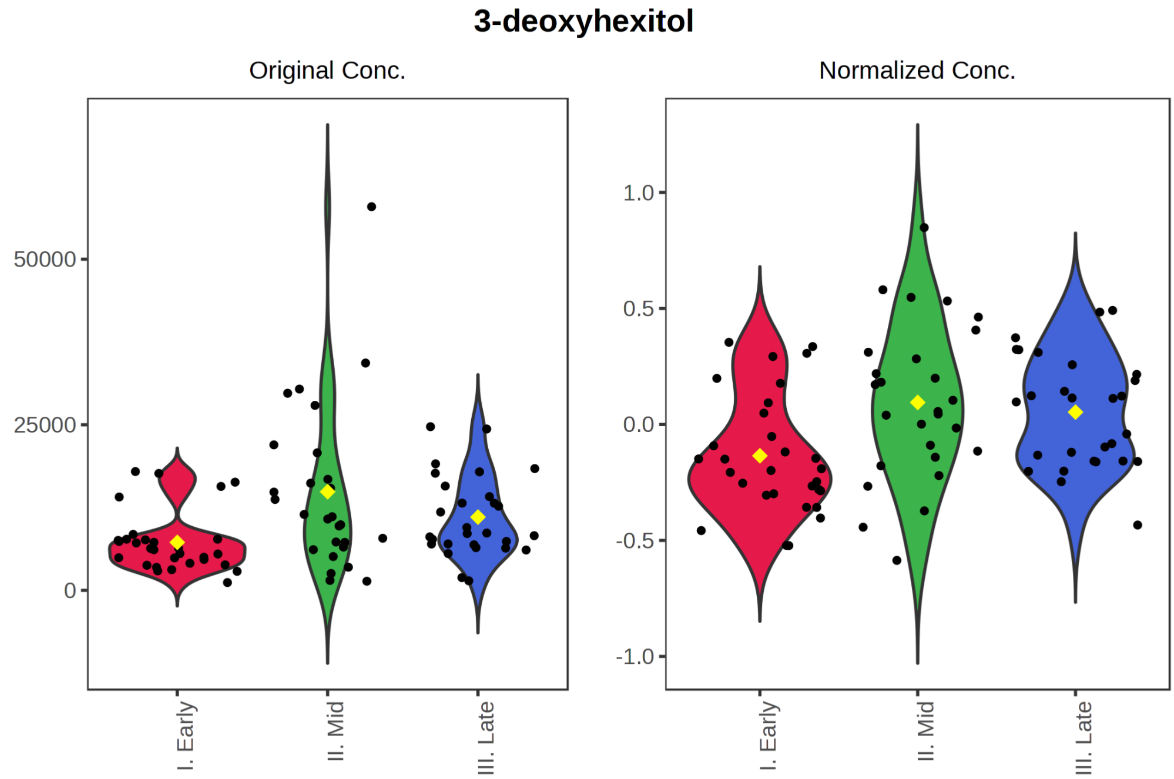
3-deoxyhexitol
Original Conc.
Normalized Conc.
50000
25000
0
1.0
0.5
0.0
-0.5
-1.0
I. Early
II. Mid
III. Late

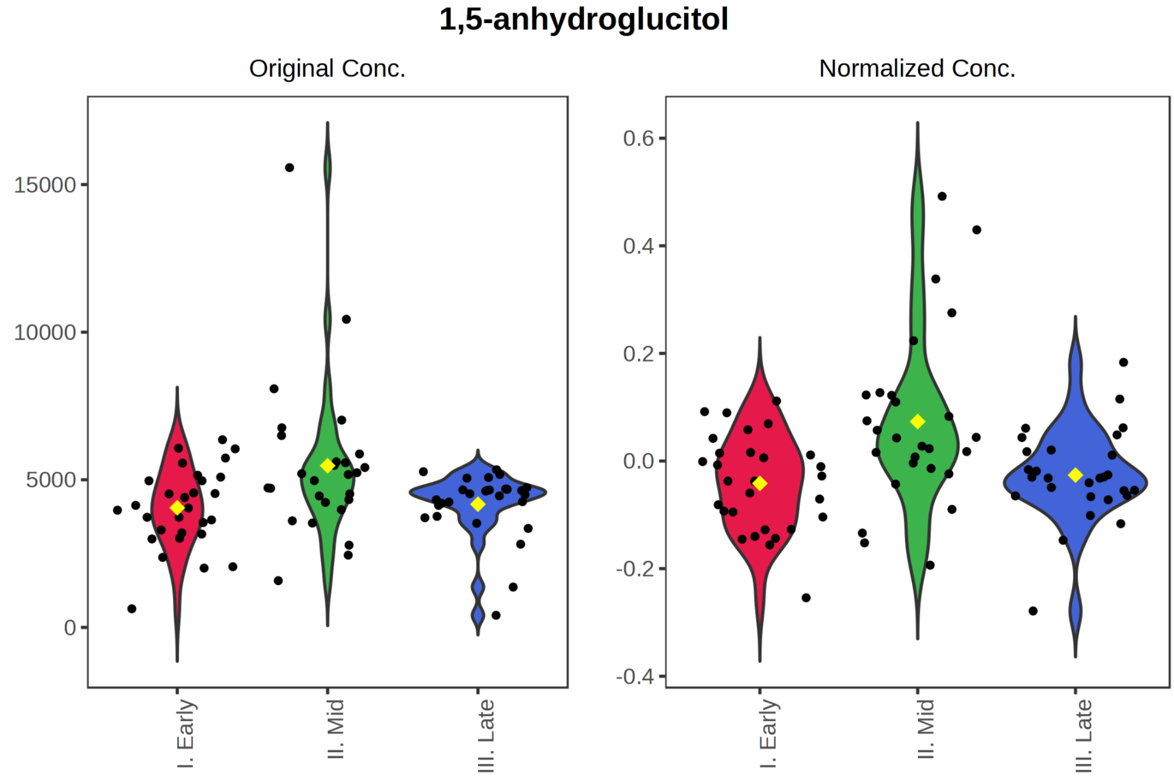
1,5-anhydroglucitol
Original Conc.
Normalized Conc.
15000
10000
5000
0
0.6
0.4
0.2
0.0
-0.2
-0.4
I. Early
II. Mid
III. Late

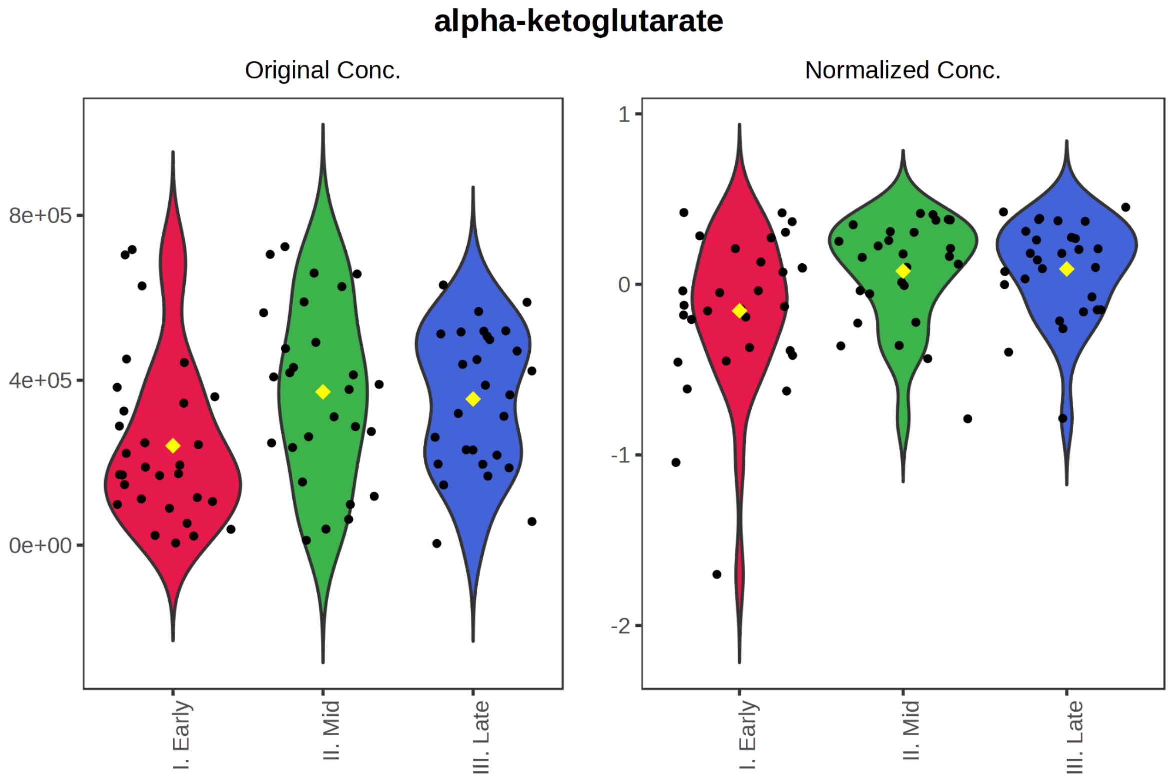
alpha-ketoglutarate
Original Conc.
Normalized Conc.
8e+05
4e+05
0e+00
1
0
-1
-2
I. Early
II. Mid
III. Late

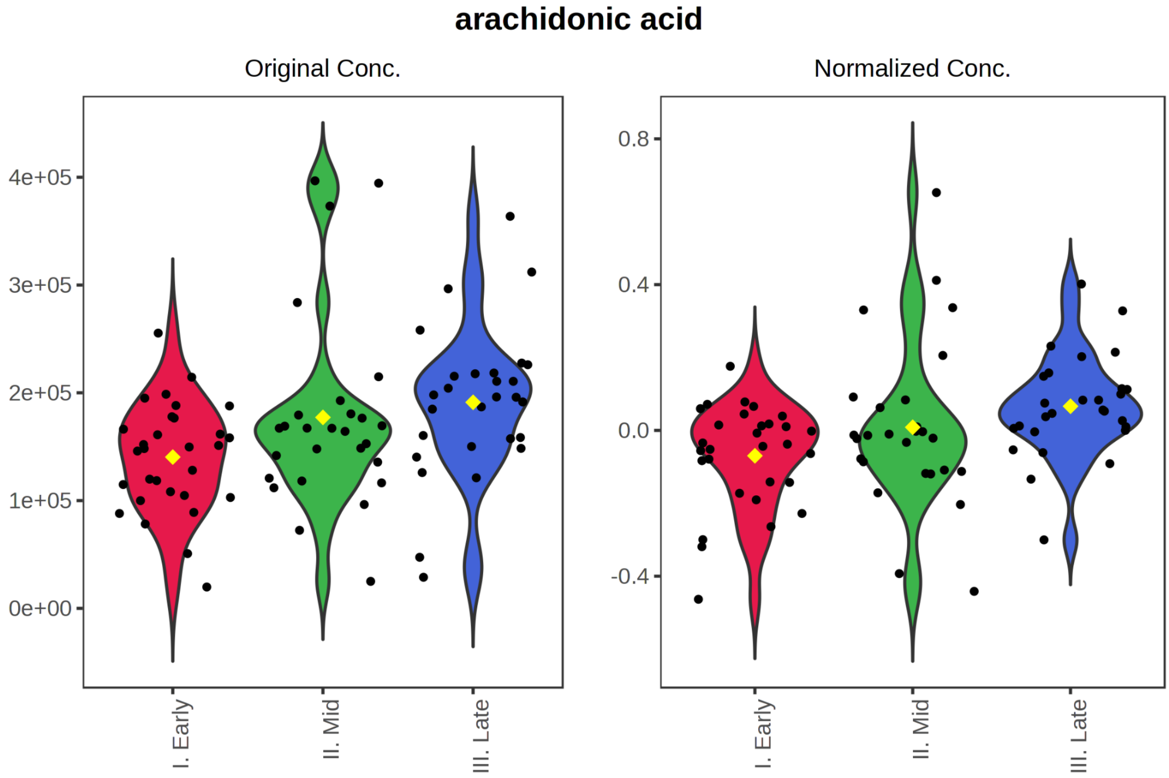
arachidonic acid
Original Conc.
Normalized Conc.
4e+05
3e+05
2e+05
1e+05
0e+00
0.8
0.4
0.0
-0.4
I. Early
II. Mid
III. Late

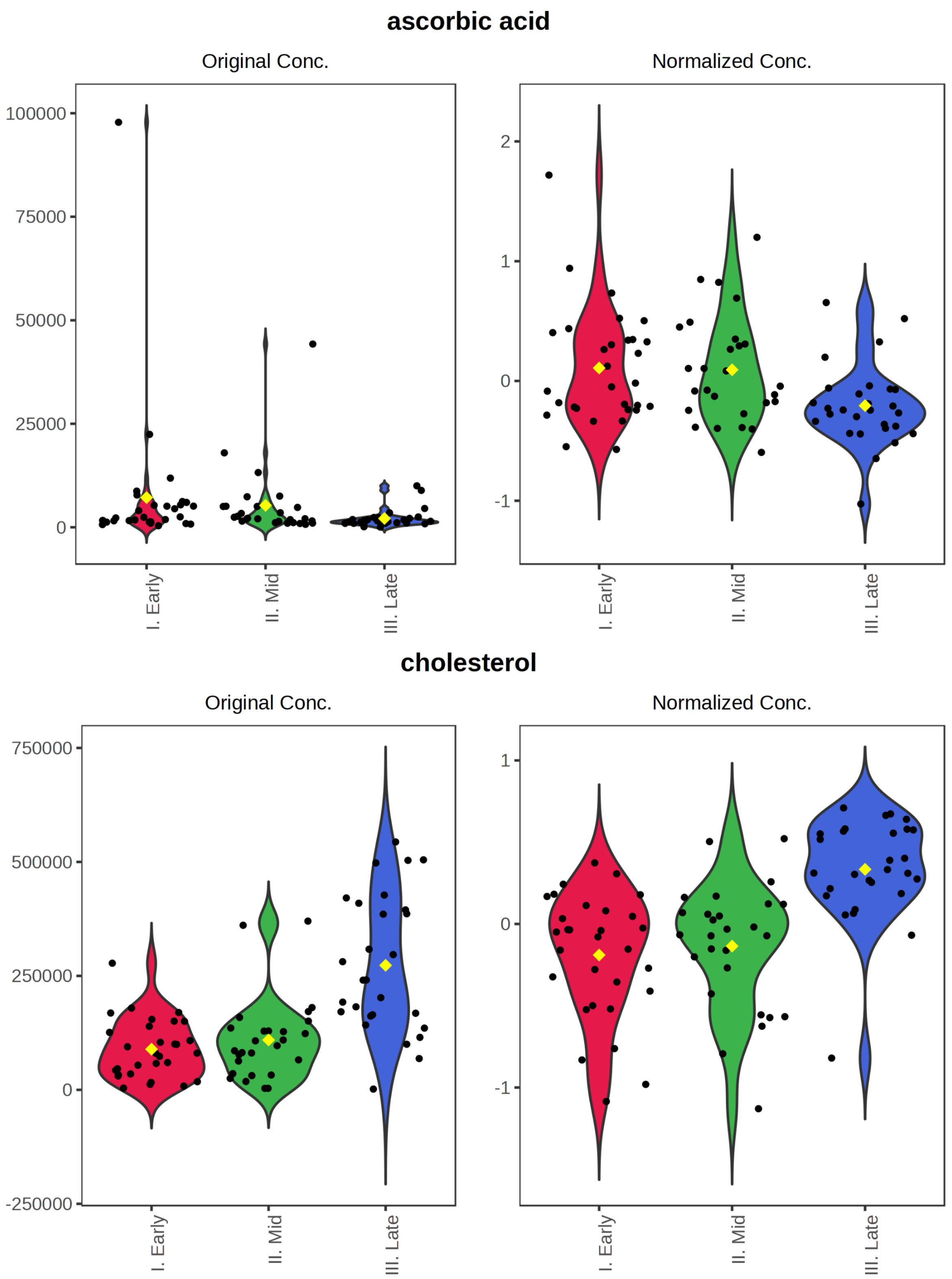
ascorbic acid
Original Conc.
Normalized Conc.
100000
75000
50000
25000
0
2
1
0
-1
I. Early
II. Mid
III. Late
cholesterol
Original Conc.
Normalized Conc.
750000
500000
250000
0
-250000
1
0
-1
I. Early
II. Mid
III. Late

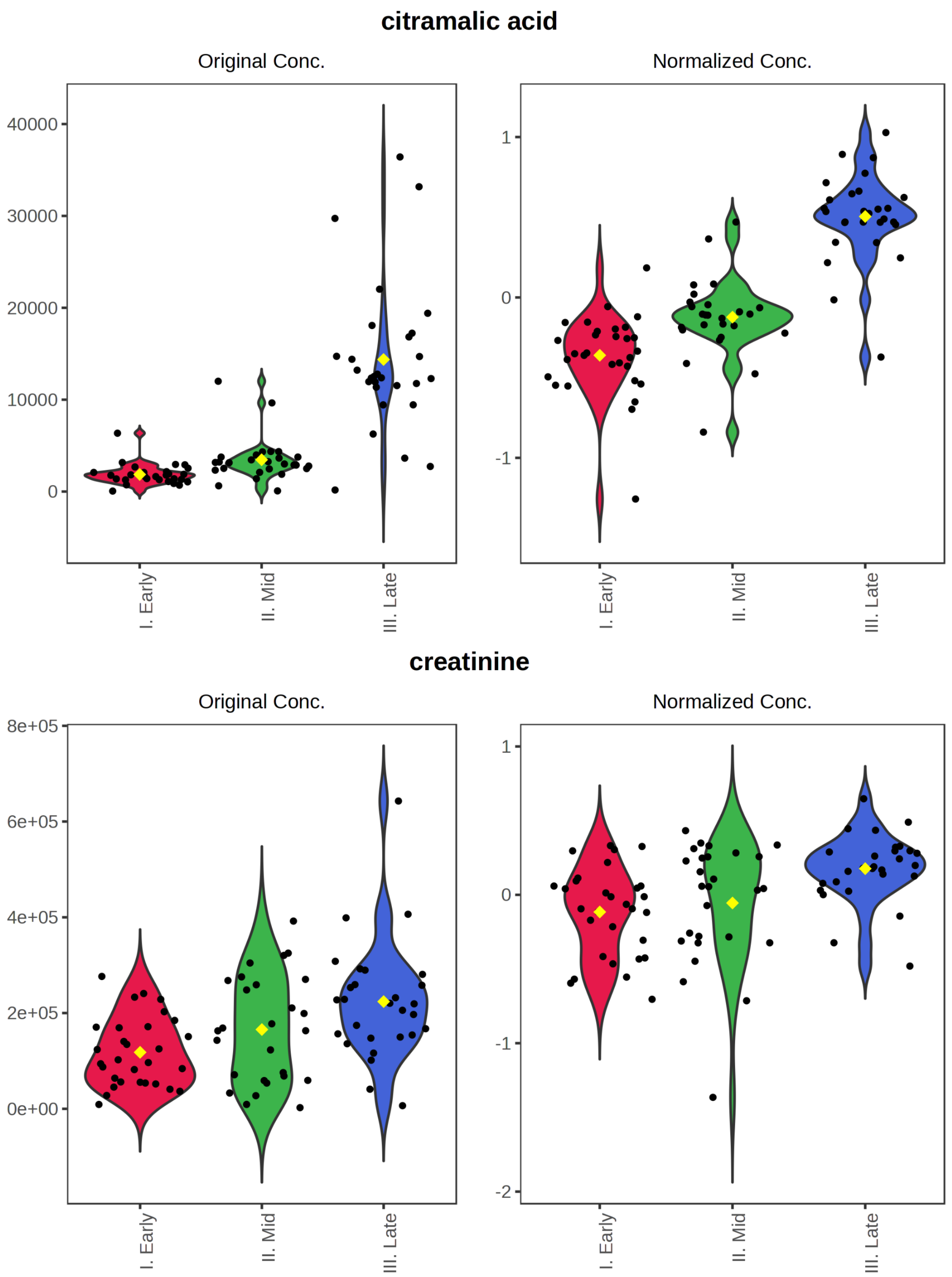

citramalic acid
Original Conc.
Normalized Conc.
40000
30000
20000
10000
0
1
0
-1
I. Early
II. Mid
III. Late
creatinine
Original Conc.
Normalized Conc.
8e+05
6e+05
4e+05
2e+05
0e+00
1
0
-1
-2
I. Early
II. Mid
III. Late

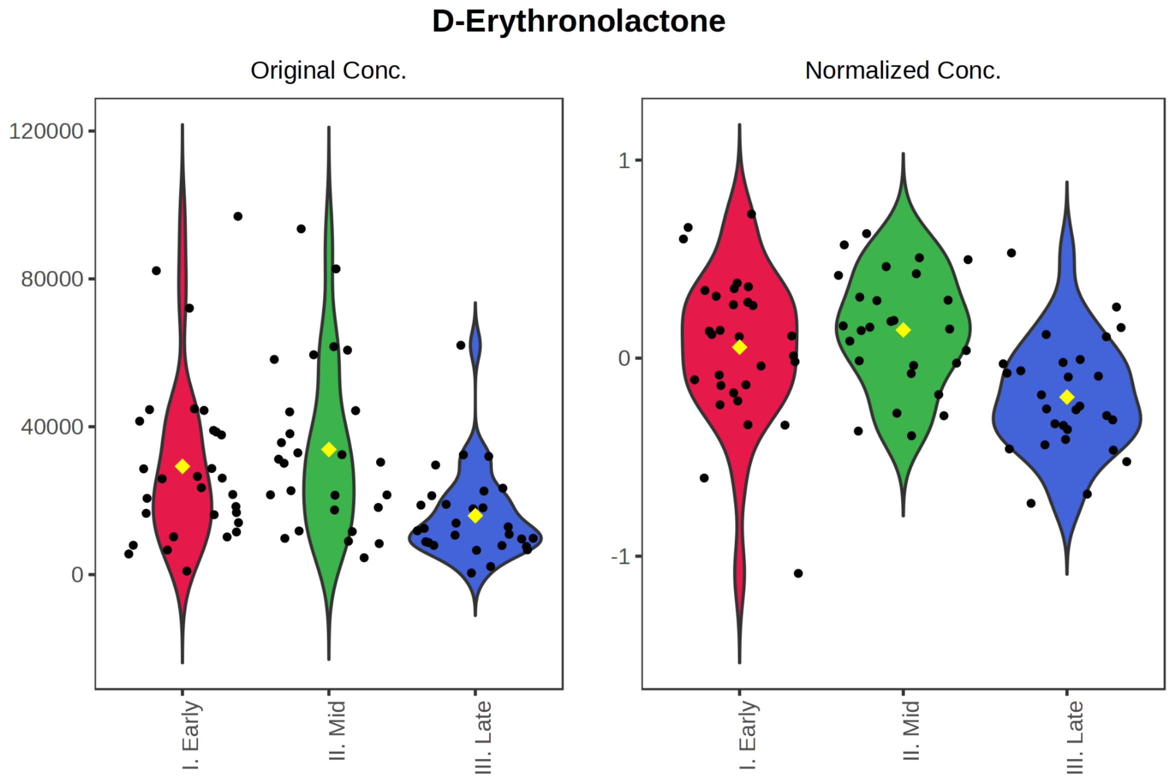
D-Erythronolactone
Original Conc.
Normalized Conc.
120000
80000
40000
0
1
0
-1
I. Early
II. Mid
III. Late

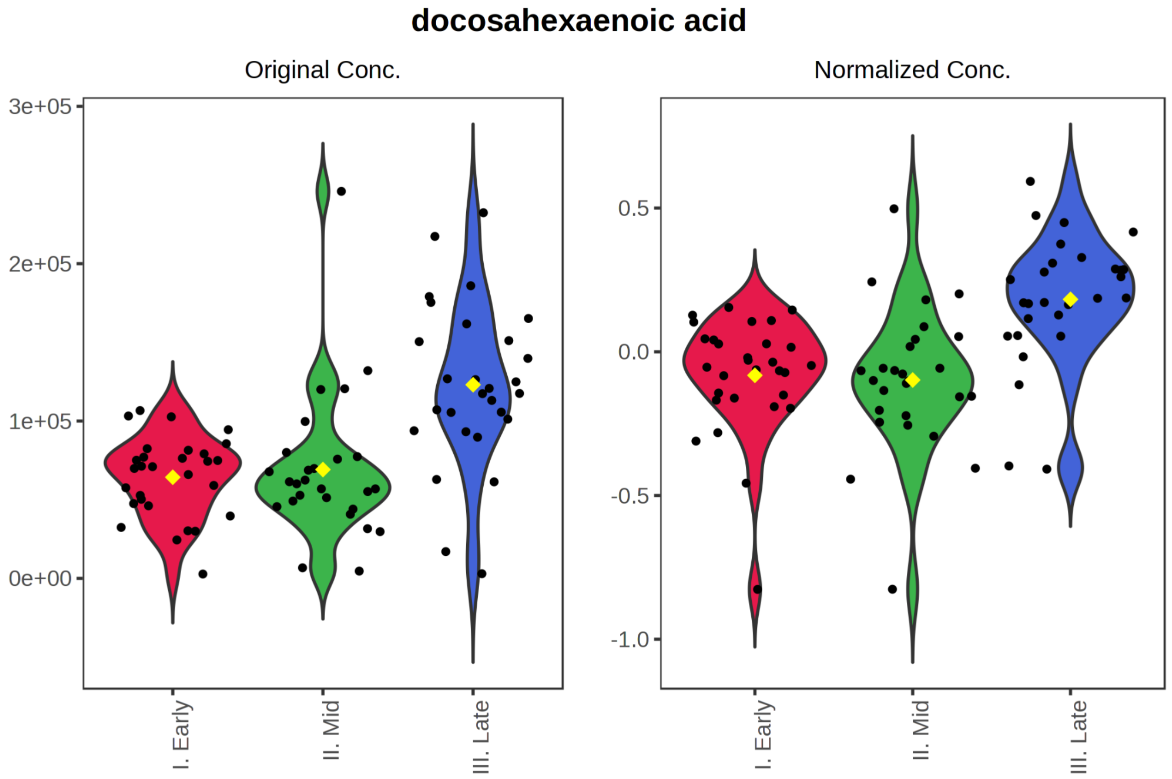
docosahexaenoic acid
Original Conc.
Normalized Conc.
3e+05
2e+05
1e+05
0e+00
0.5
0.0
-0.5
-1.0
I. Early
II. Mid
III. Late

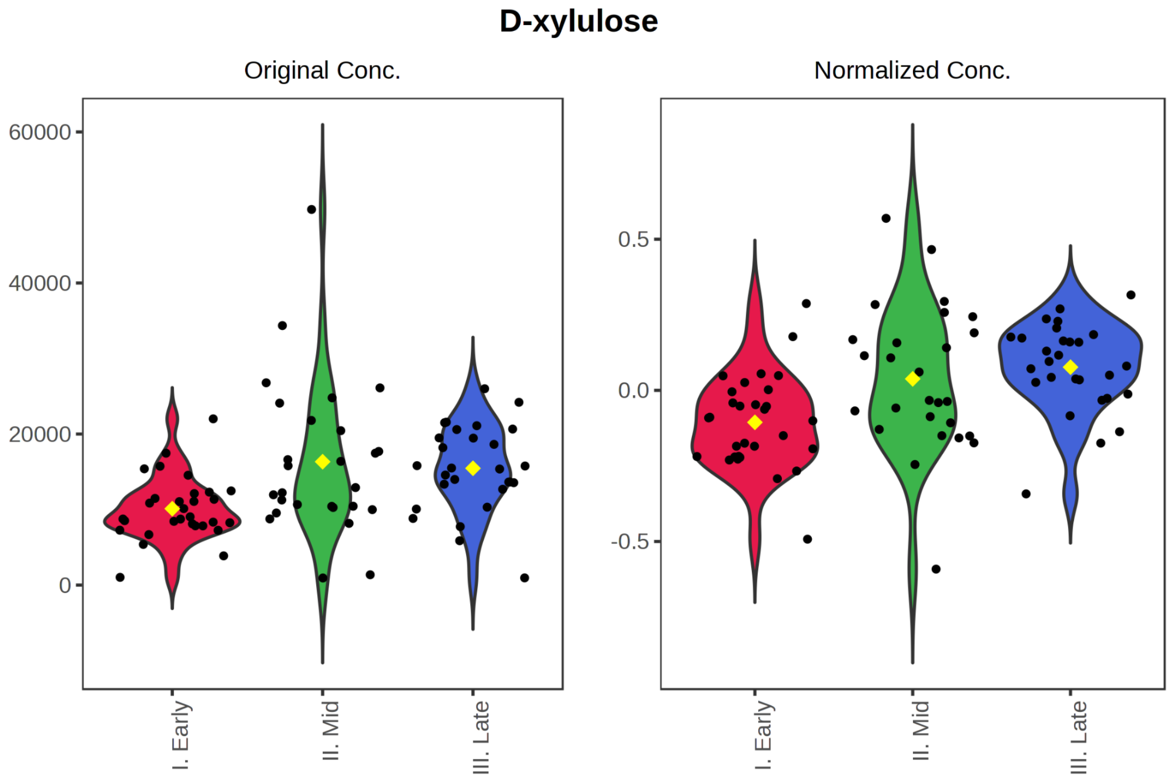
D-xylulose
Original Conc.
Normalized Conc.
60000
40000
20000
0
0.5
0.0
-0.5
I. Early
II. Mid
III. Late

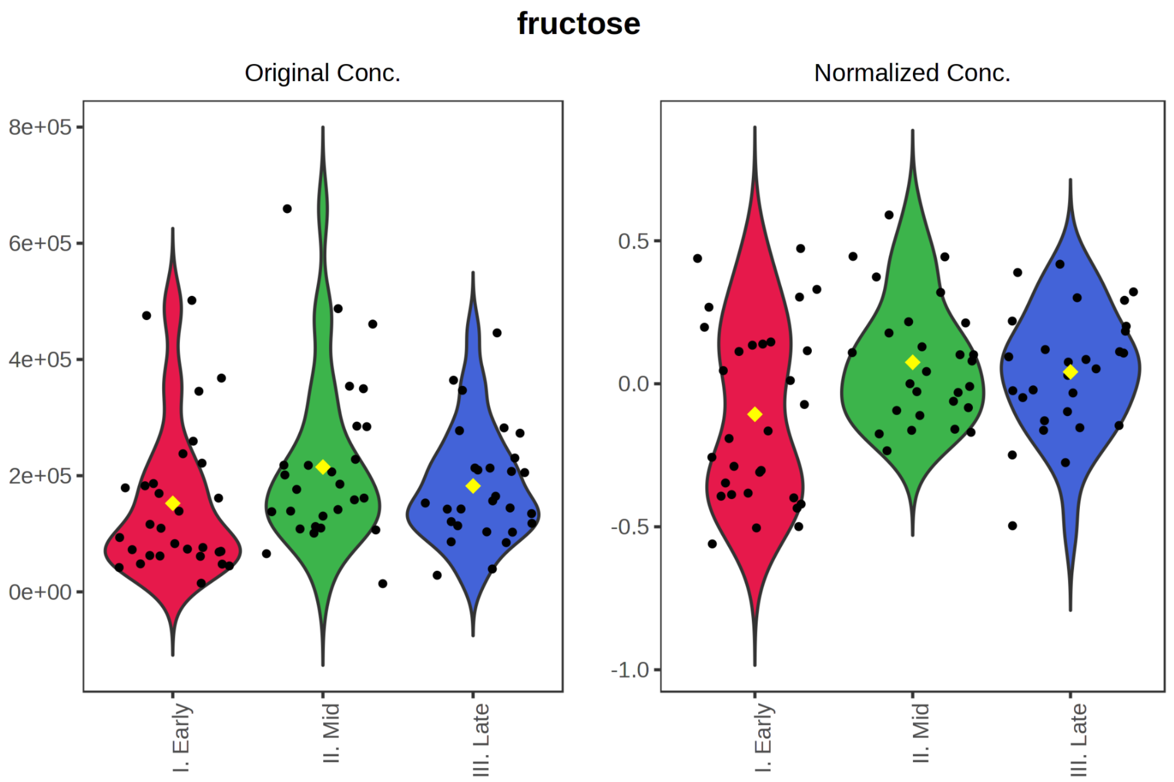
fructose
Original Conc.
Normalized Conc.
8e+05
6e+05
4e+05
2e+05
0e+00
0.5
0.0
-0.5
-1.0
I. Early
II. Mid
III. Late

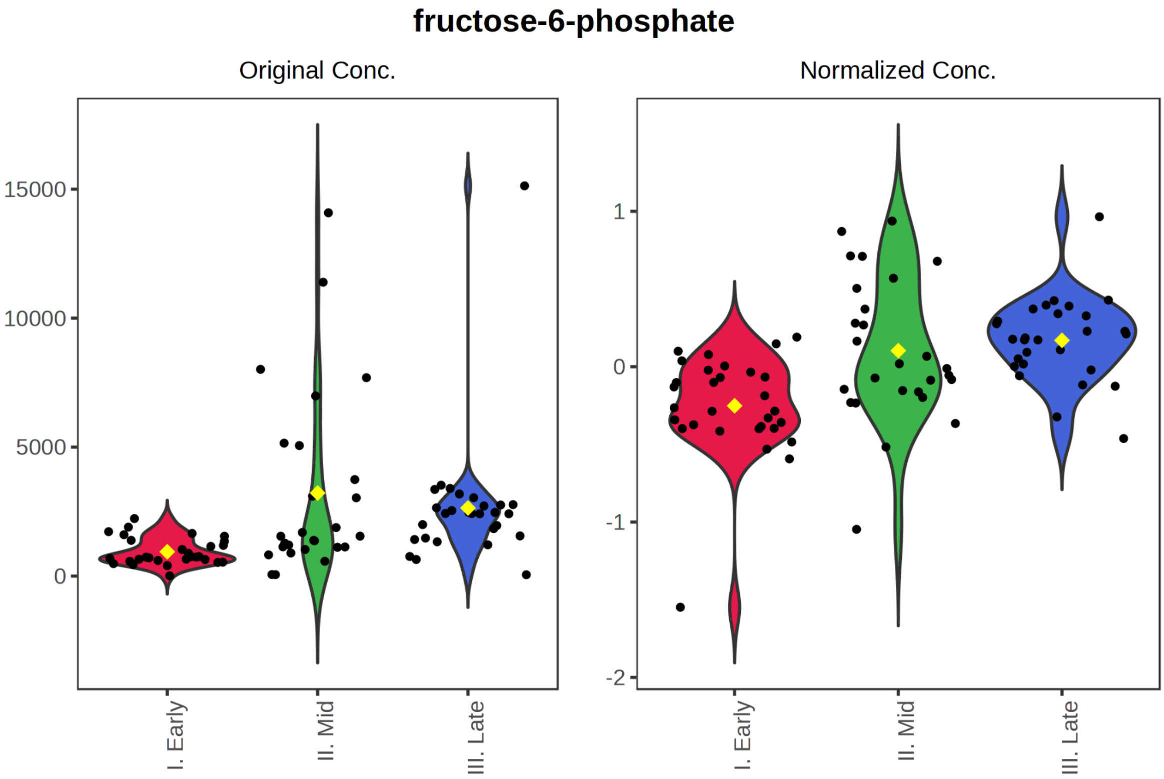
fructose-6-phosphate
Original Conc.
Normalized Conc.
15000
10000
5000
0
1
0
-1
-2
I. Early
II. Mid
III. Late

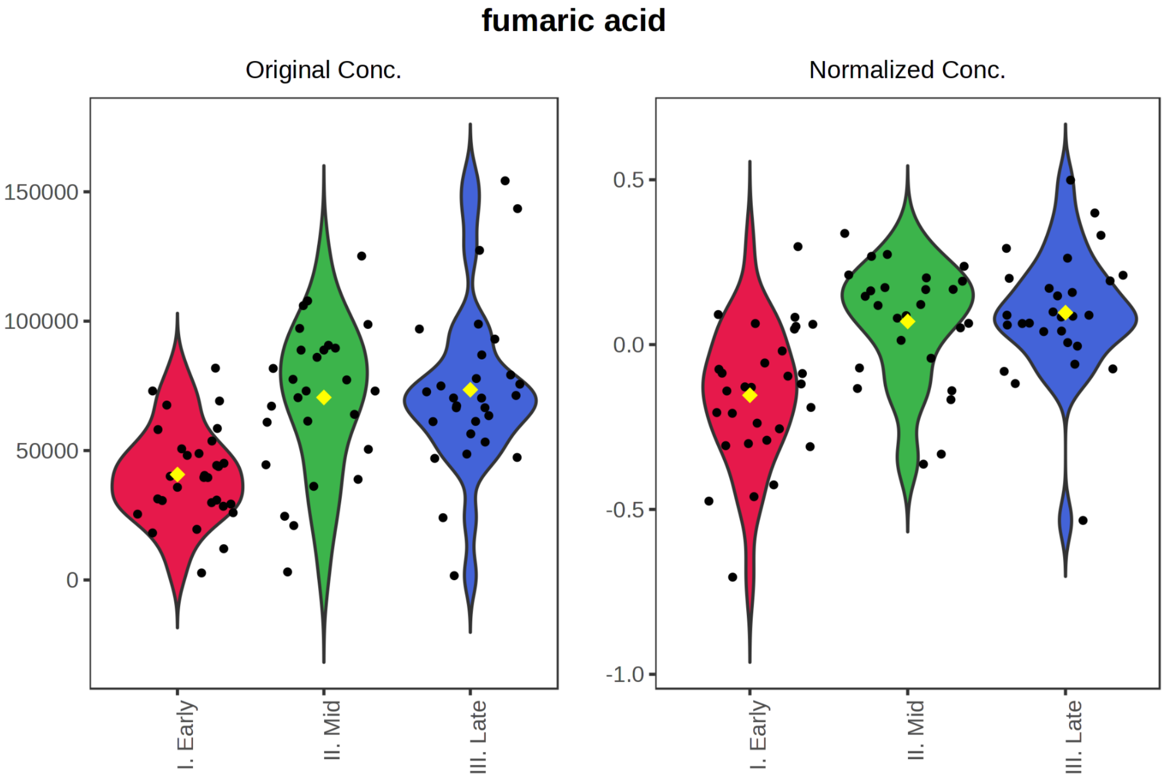
fumaric acid
Original Conc.
Normalized Conc.
150000
100000
50000
0
0.5
0.0
-0.5
-1.0
I. Early
II. Mid
III. Late

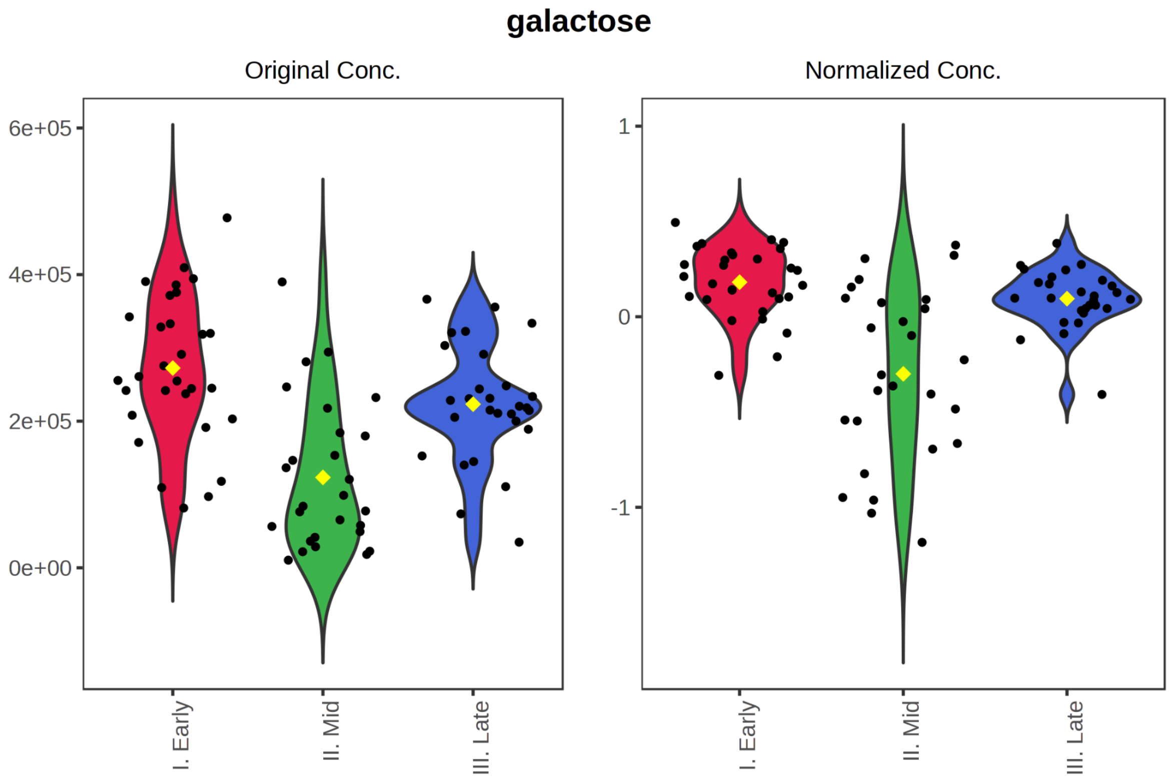

galactose
Original Conc.
Normalized Conc.
6e+05
4e+05
2e+05
0e+00
1
0
-1
I. Early
II. Mid
III. Late

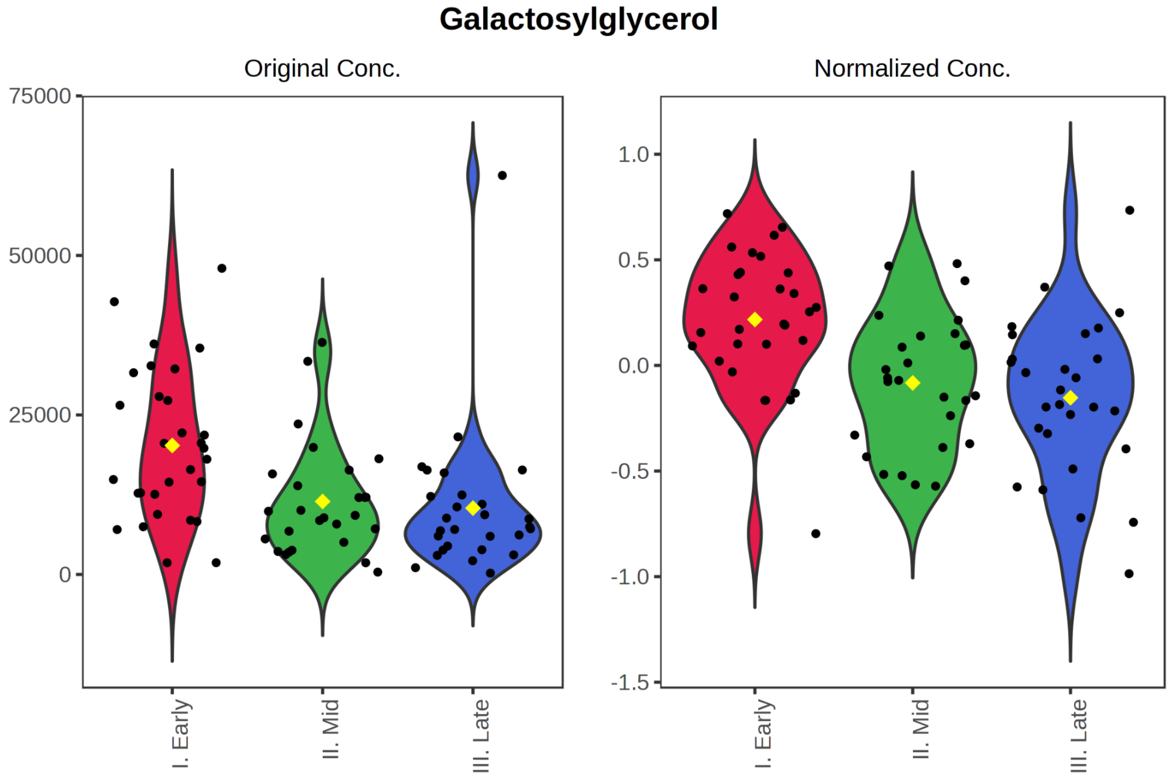

Galactosylglycerol
Original Conc.
Normalized Conc.
75000
50000
25000
0
1.0
0.5
0.0
-0.5
-1.0
-1.5
I. Early
II. Mid
III. Late

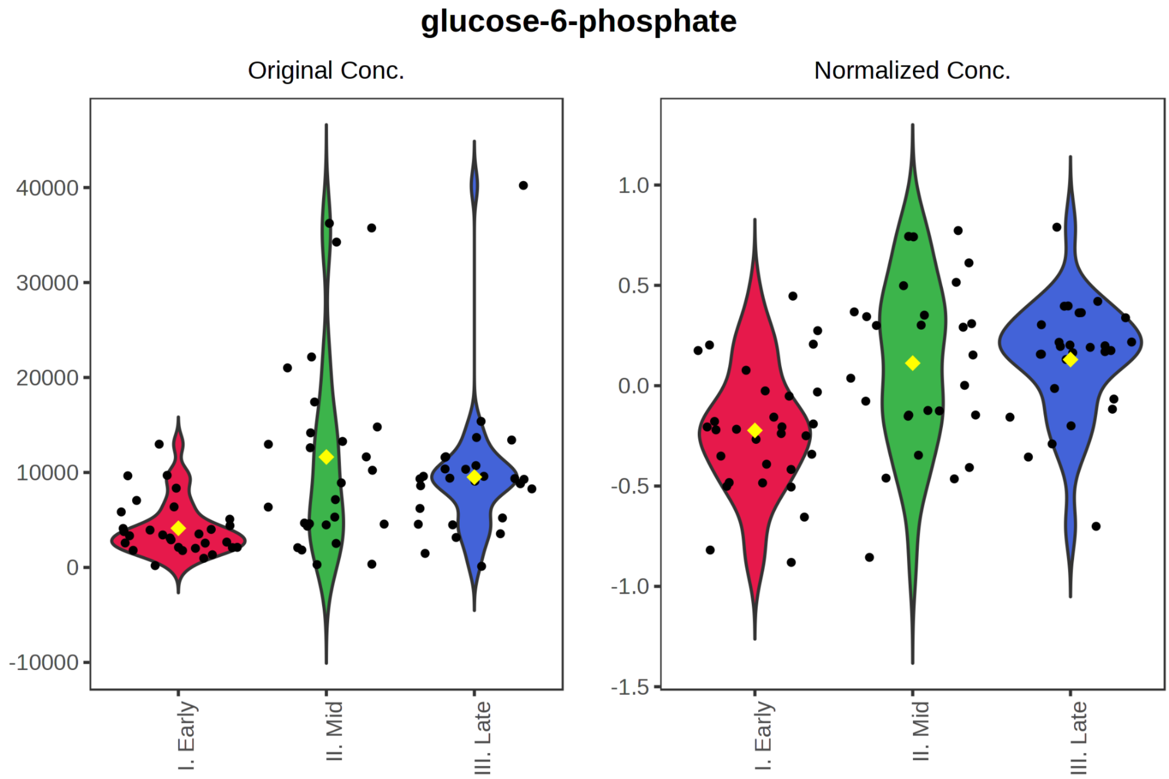

glucose-6-phosphate
Original Conc.
Normalized Conc.
40000
30000
20000
10000
0
-10000
1.0
0.5
0.0
-0.5
-1.0
-1.5
I. Early
II. Mid
III. Late

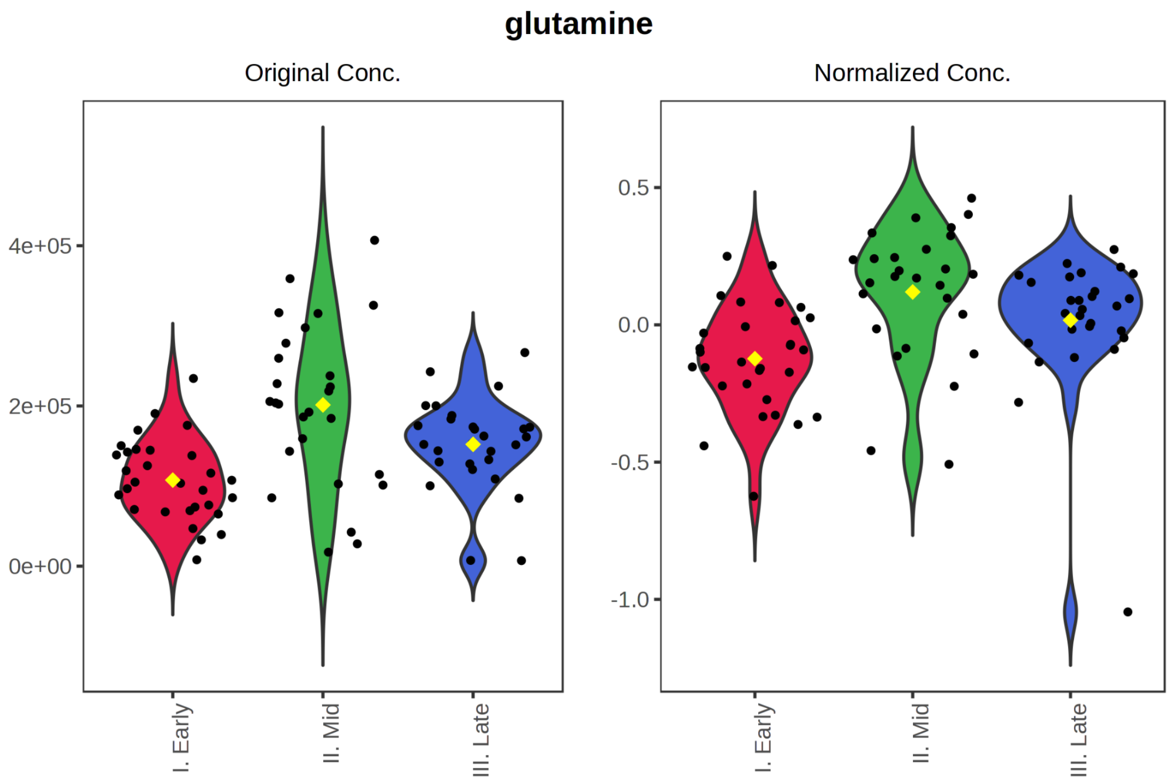

glutamine
Original Conc.
Normalized Conc.
4e+05
2e+05
0e+00
0.5
0.0
-0.5
-1.0
I. Early
II. Mid
III. Late

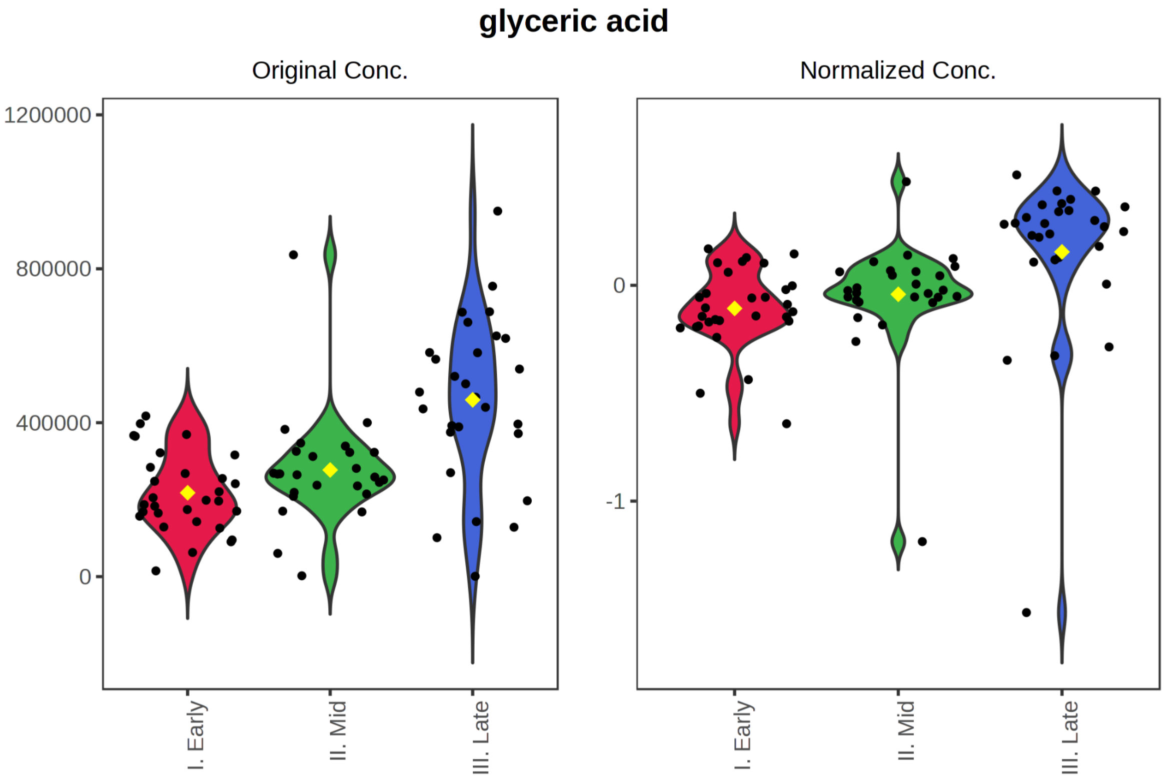
glyceric acid
Original Conc.
Normalized Conc.
1200000
800000
400000
0
-1
I. Early
II. Mid
III. Late

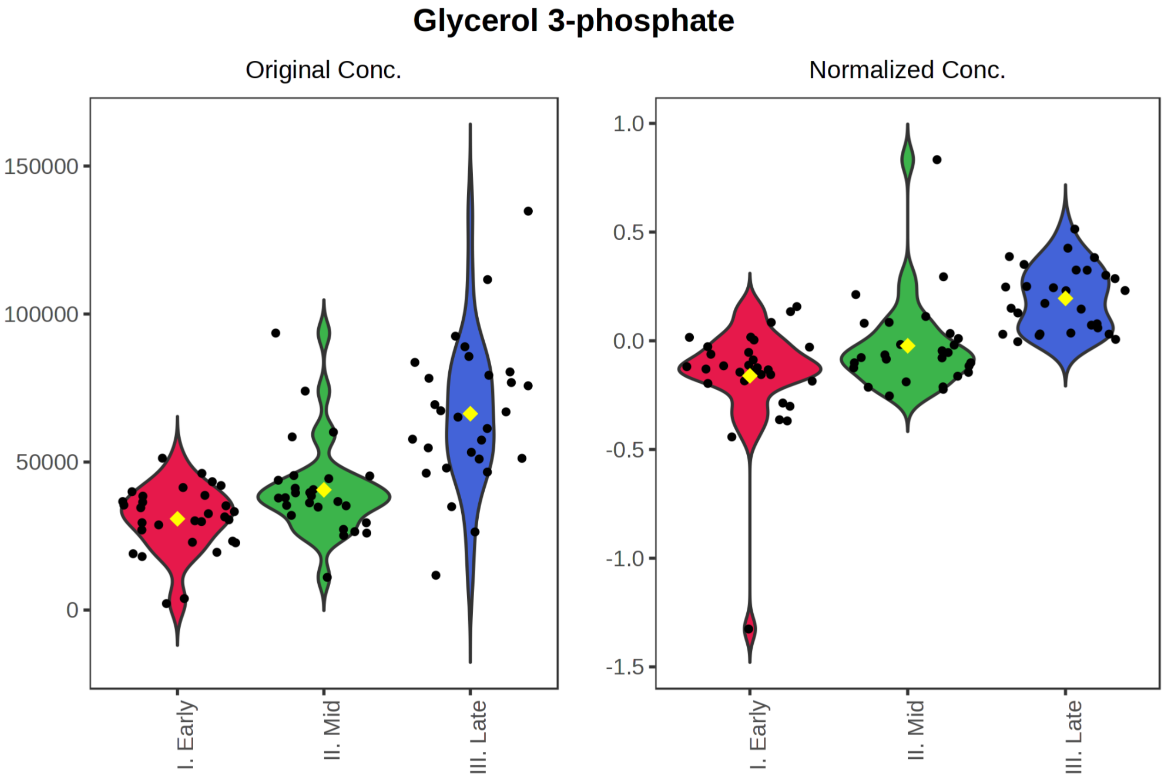
Glycerol 3-phosphate
Original Conc.
Normalized Conc.
150000
100000
50000
0
1.0
0.5
0.0
-0.5
-1.0
-1.5
I. Early
II. Mid
III. Late

Glyceryl 2-myristate

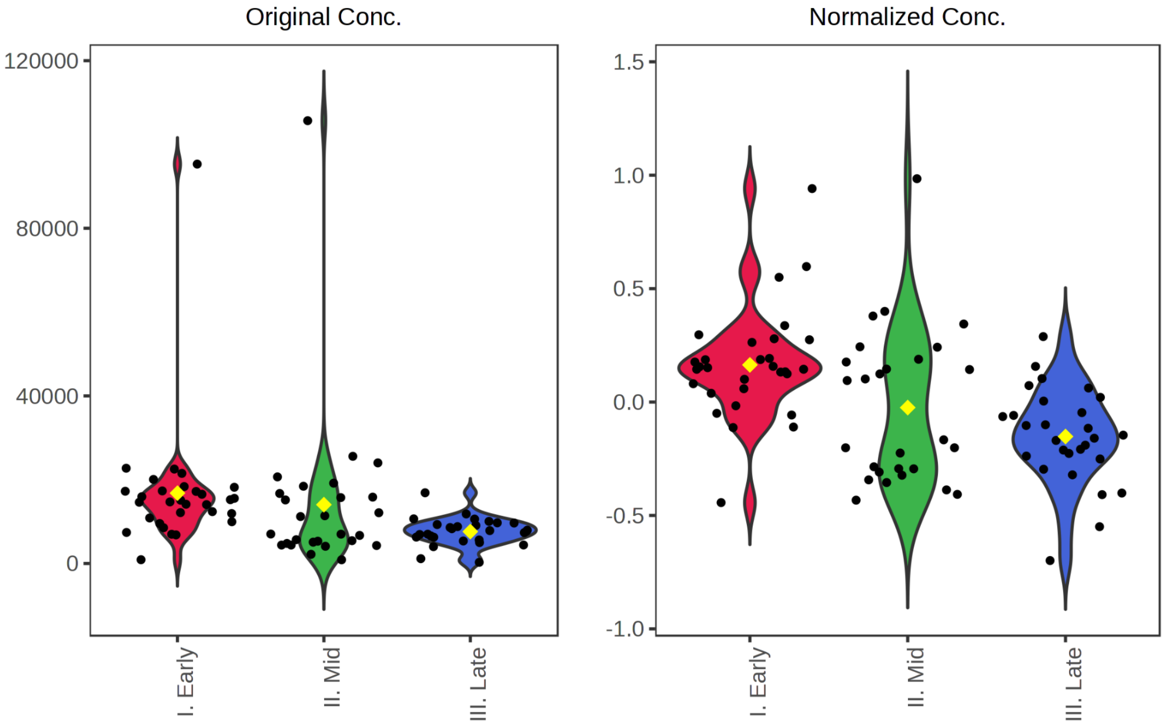

glyceryl monostearate

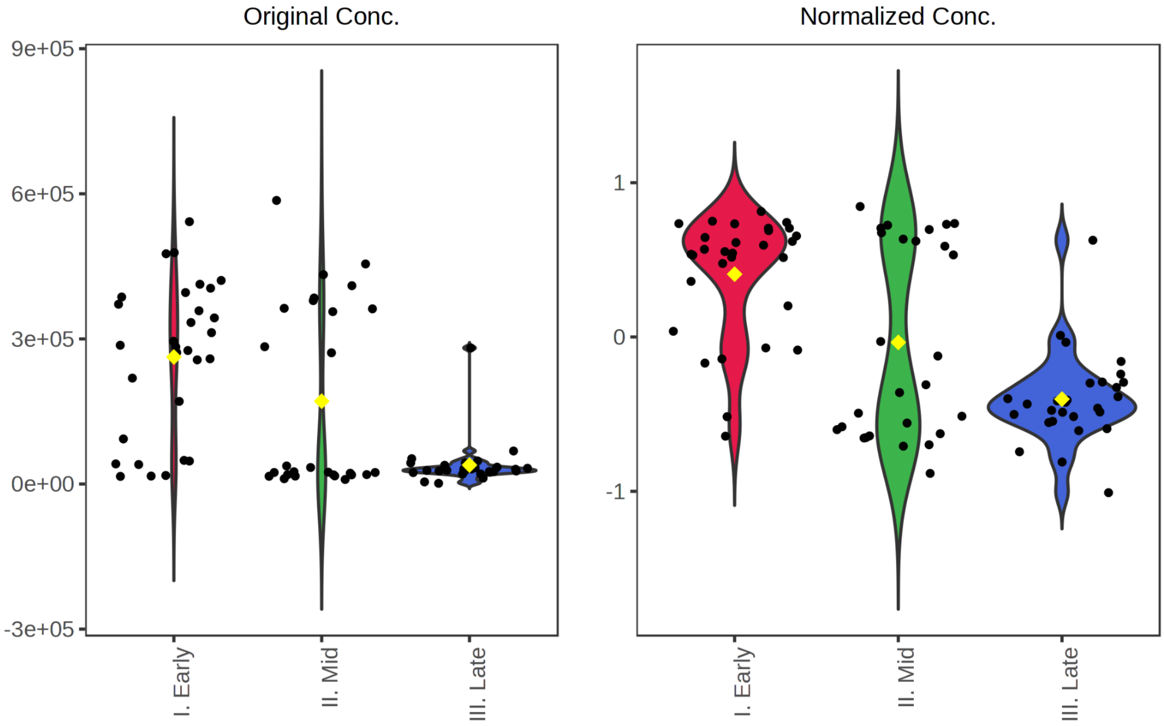

hexitol

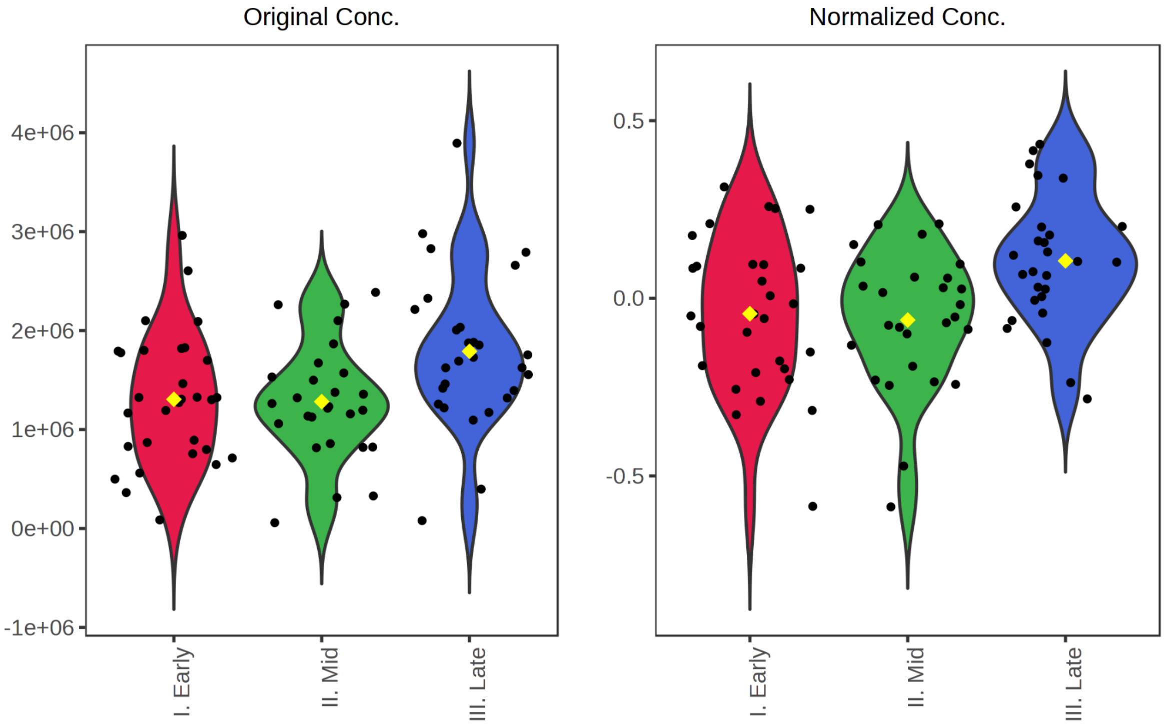

Inositol 4-phosphate

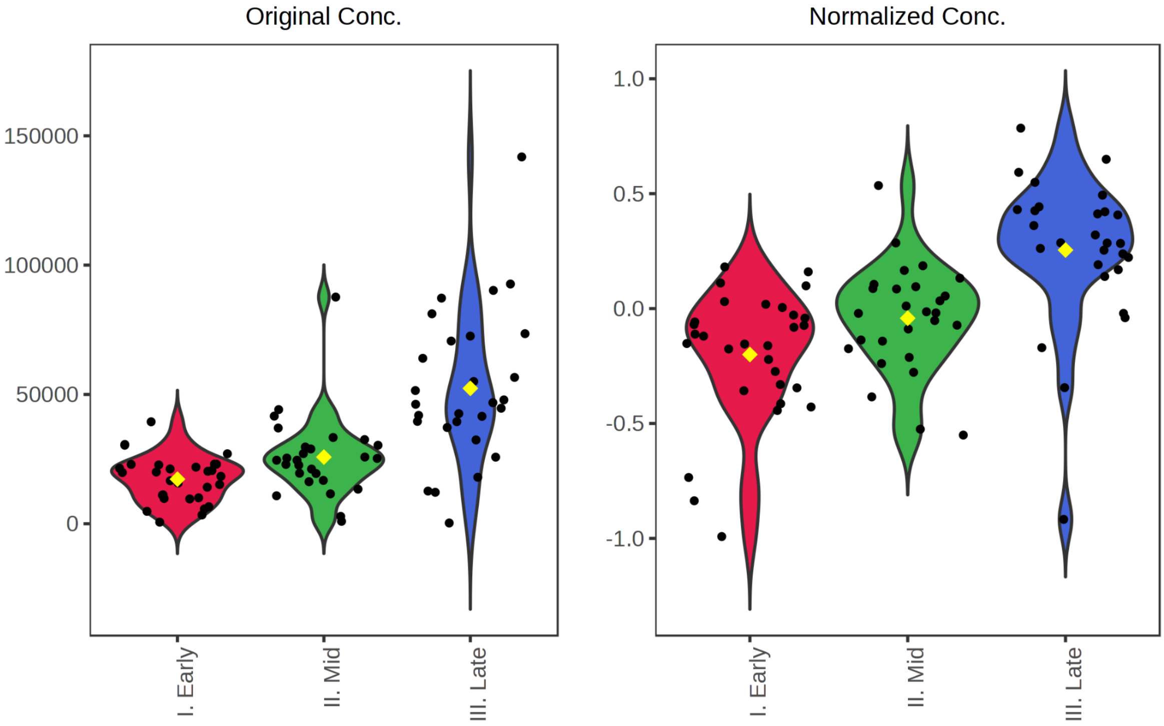

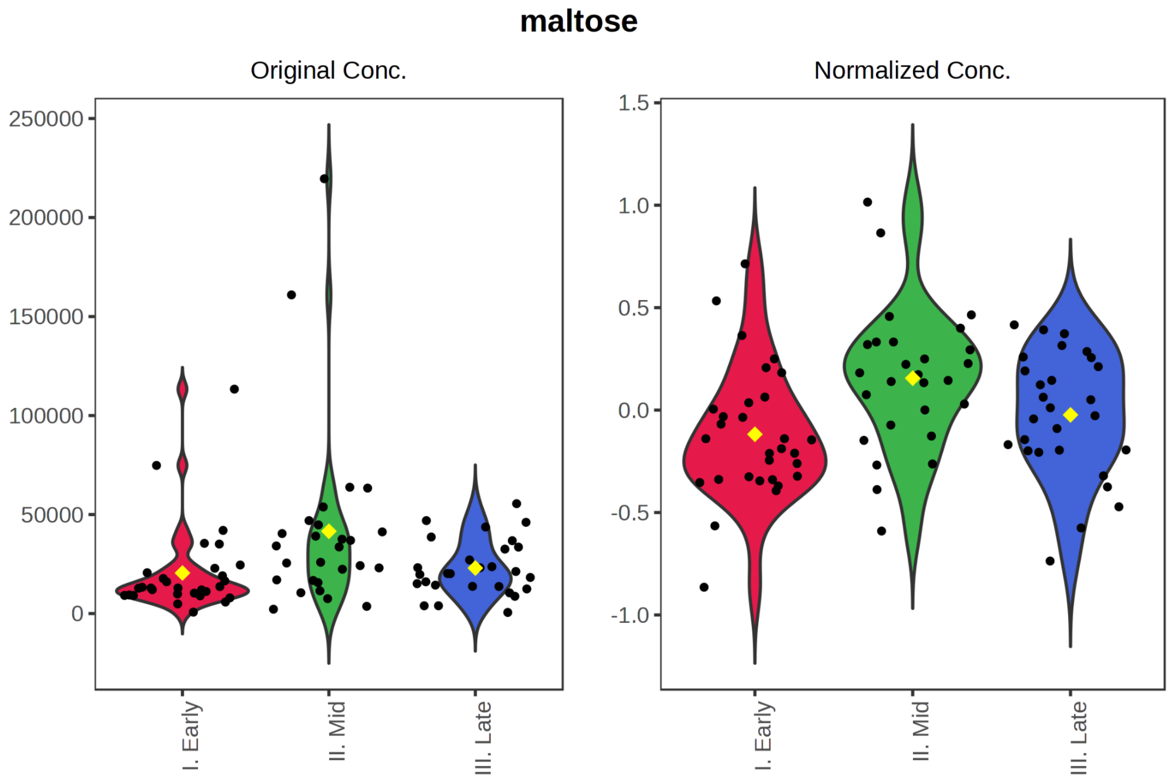
maltose
Original Conc.
Normalized Conc.
250000
200000
150000
100000
50000
0
1.5
1.0
0.5
0.0
-0.5
-1.0
I. Early
II. Mid
III. Late

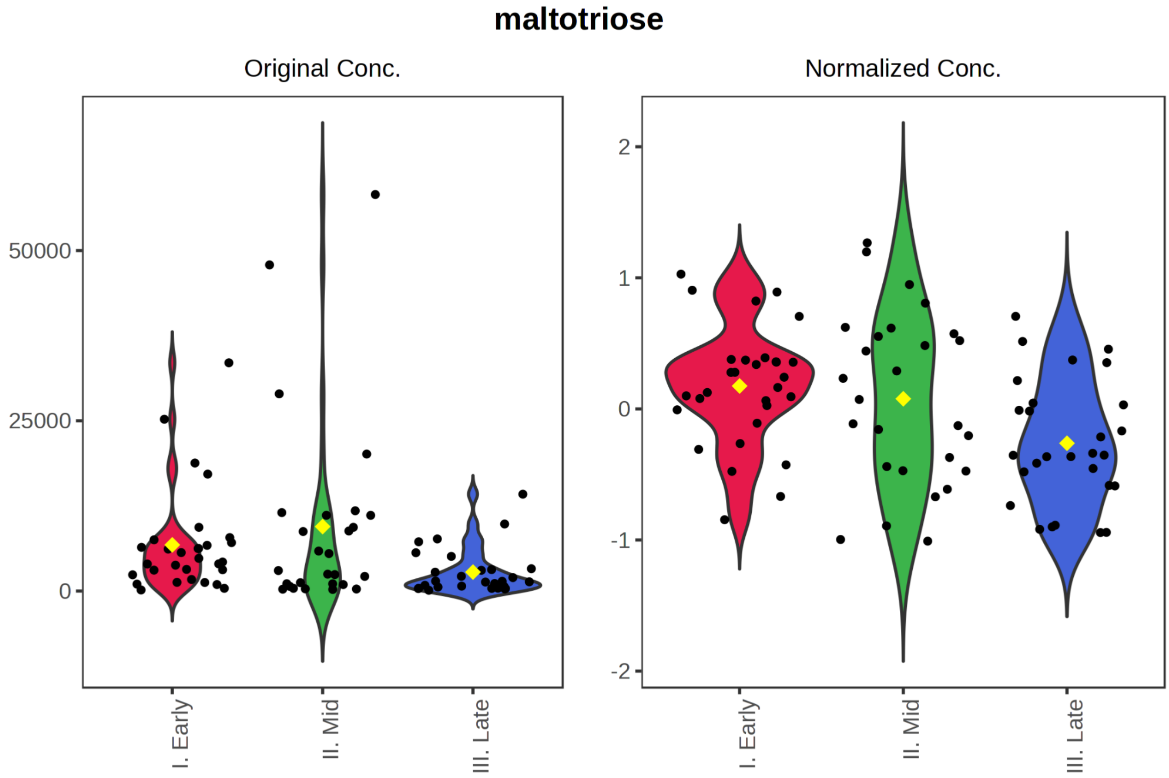
maltotriose
Original Conc.
Normalized Conc.
50000
25000
0
2
1
0
-1
-2
I. Early
II. Mid
III. Late

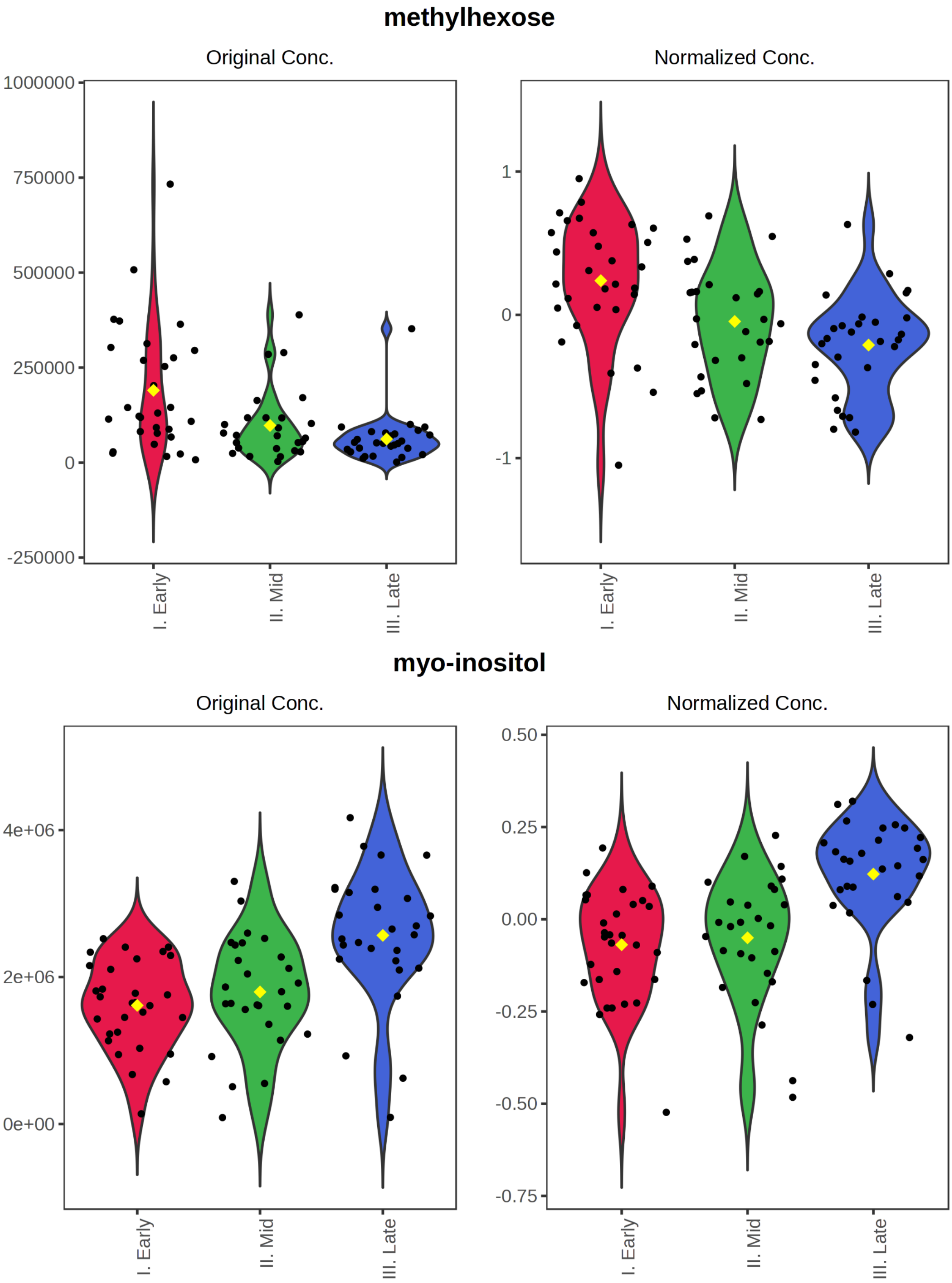
methylhexose
Original Conc.
Normalized Conc.
1000000
750000
500000
250000
0
-250000
1
0
-1
I. Early
II. Mid
III. Late
myo-inositol
Original Conc.
Normalized Conc.
4e+06
2e+06
0e+00
0.50
0.25
0.00
-0.25
-0.50
-0.75

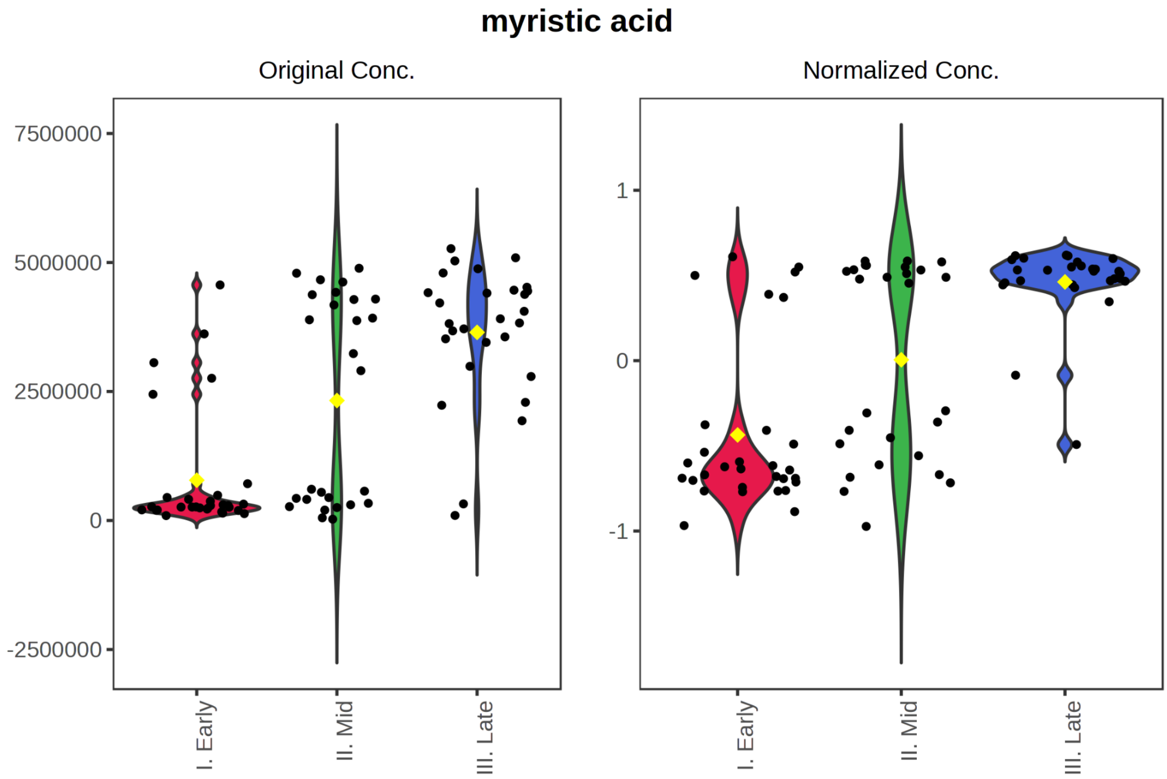
myristic acid
Original Conc.
Normalized Conc.
7500000
5000000
2500000
0
-2500000
1
0
-1
I. Early
II. Mid
III. Late
I. Early
II. Mid
III. Late

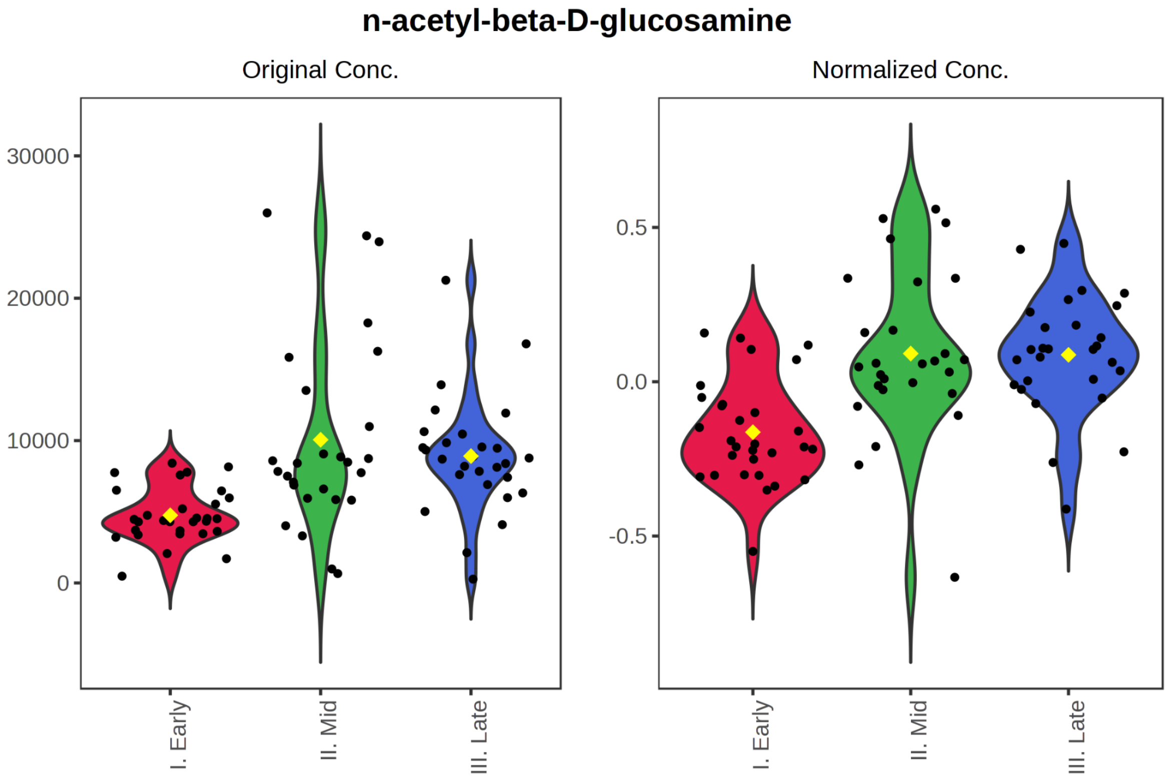
n-acetyl-beta-D-glucosamine
Original Conc.
Normalized Conc.
30000
20000
10000
0
0.5
0.0
-0.5
I. Early
II. Mid
III. Late
I. Early
II. Mid
III. Late

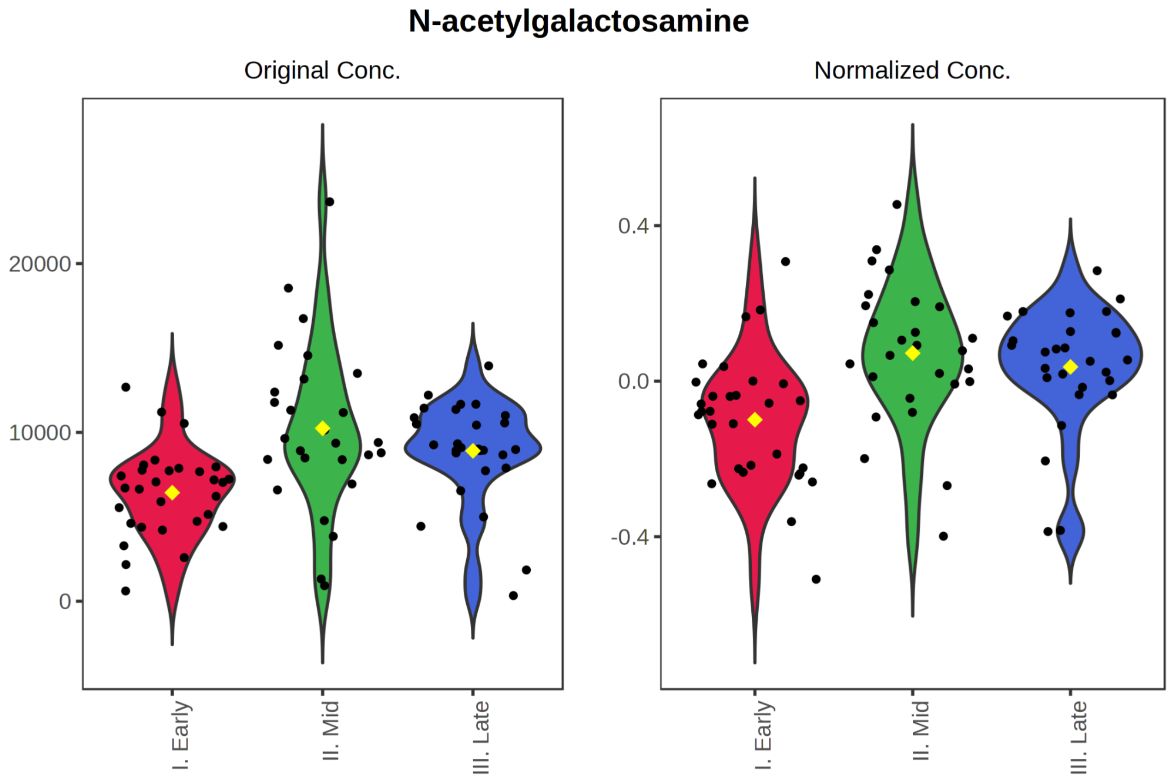
N-acetylgalactosamine
Original Conc.
Normalized Conc.
20000
10000
0
0.4
0.0
-0.4
I. Early
II. Mid
III. Late

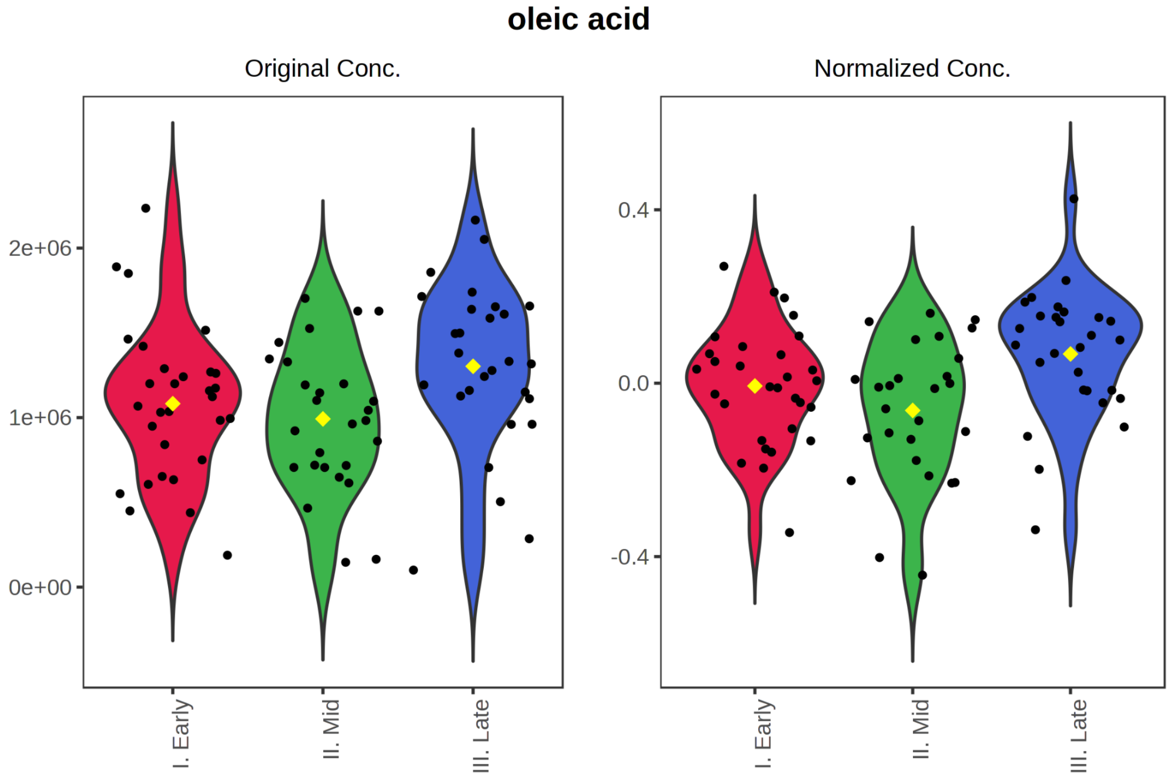
oleic acid
Original Conc.
Normalized Conc.
2e+06
1e+06
0e+00
0.4
0.0
-0.4
I. Early
II. Mid
III. Late

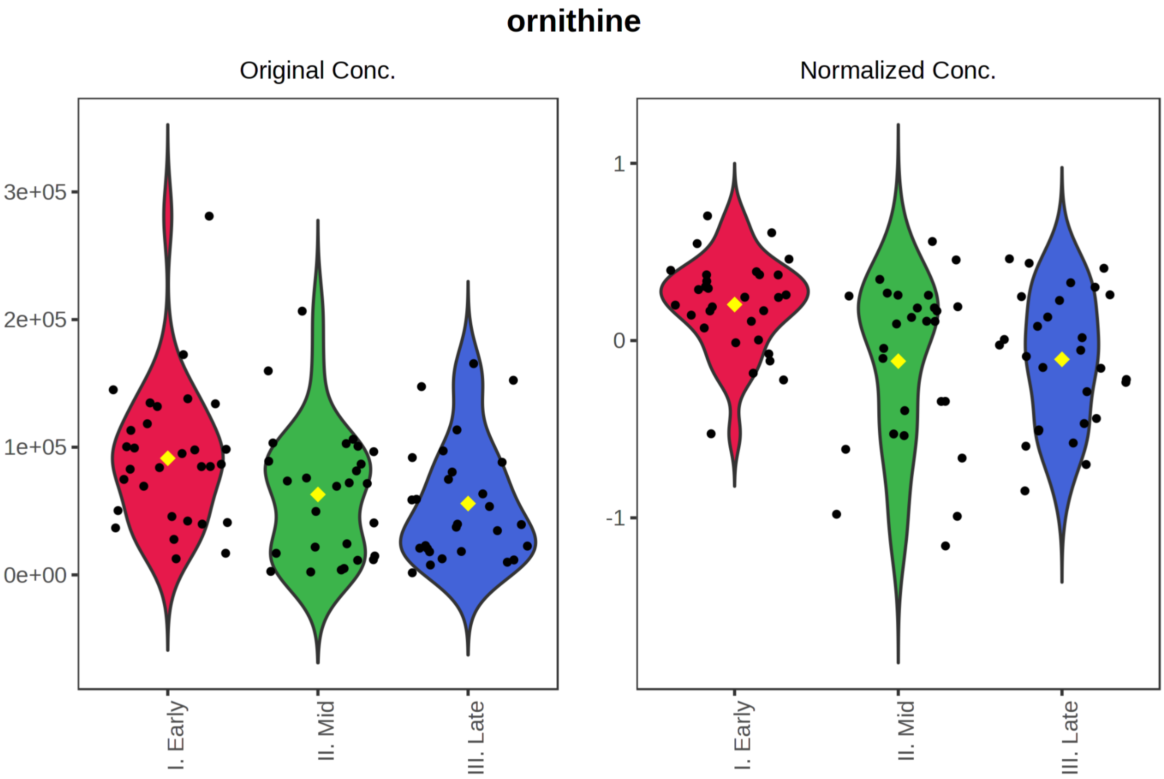
ornithine
Original Conc.
Normalized Conc.
3e+05
2e+05
1e+05
0e+00
1
0
-1
I. Early
II. Mid
III. Late

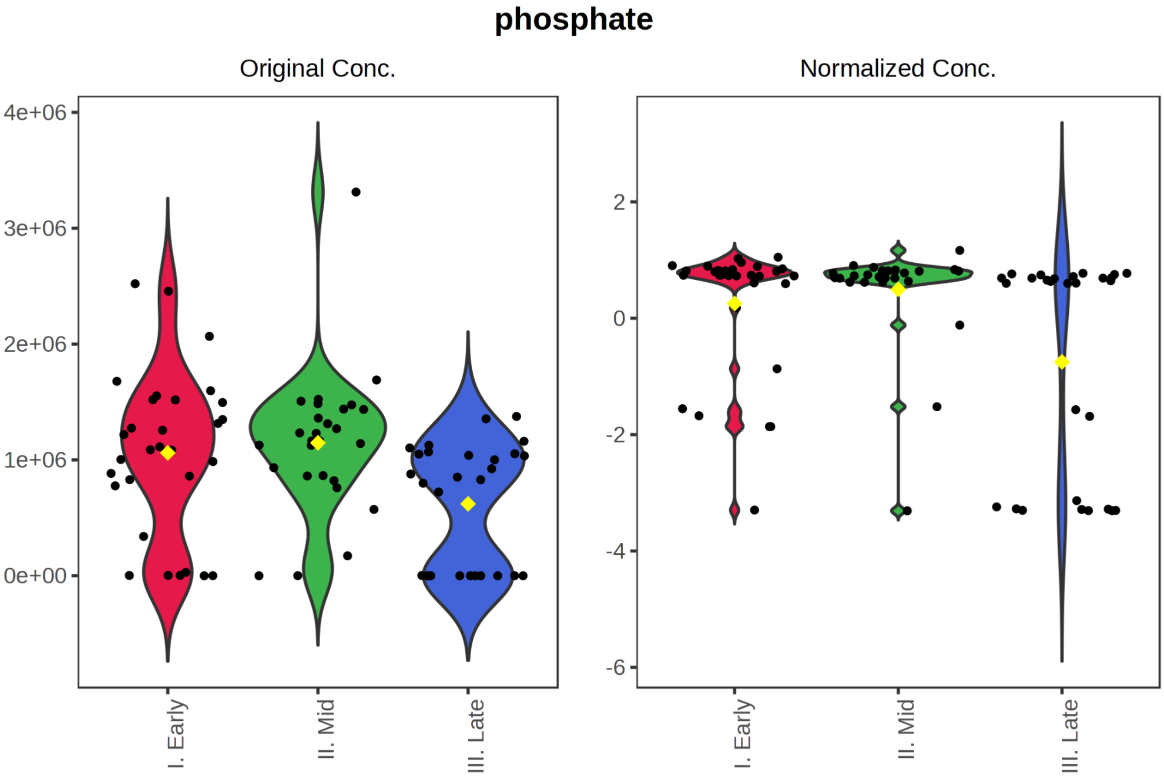
phosphate
Original Conc.
Normalized Conc.
4e+06
3e+06
2e+06
1e+06
0e+00
2
0
-2
-4
-6
I. Early
II. Mid
III. Late

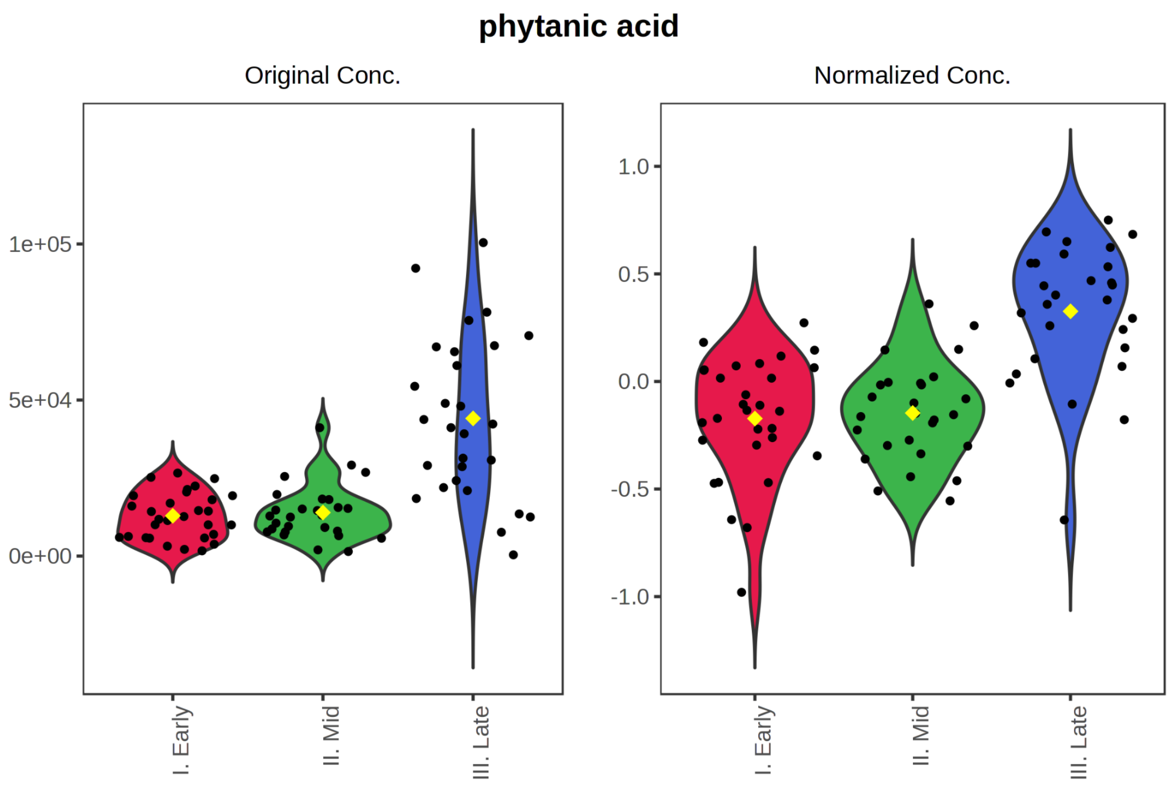
phytanic acid
Original Conc.
Normalized Conc.
1e+05
5e+04
0e+00
1.0
0.5
0.0
-0.5
-1.0
I. Early
II. Mid
III. Late

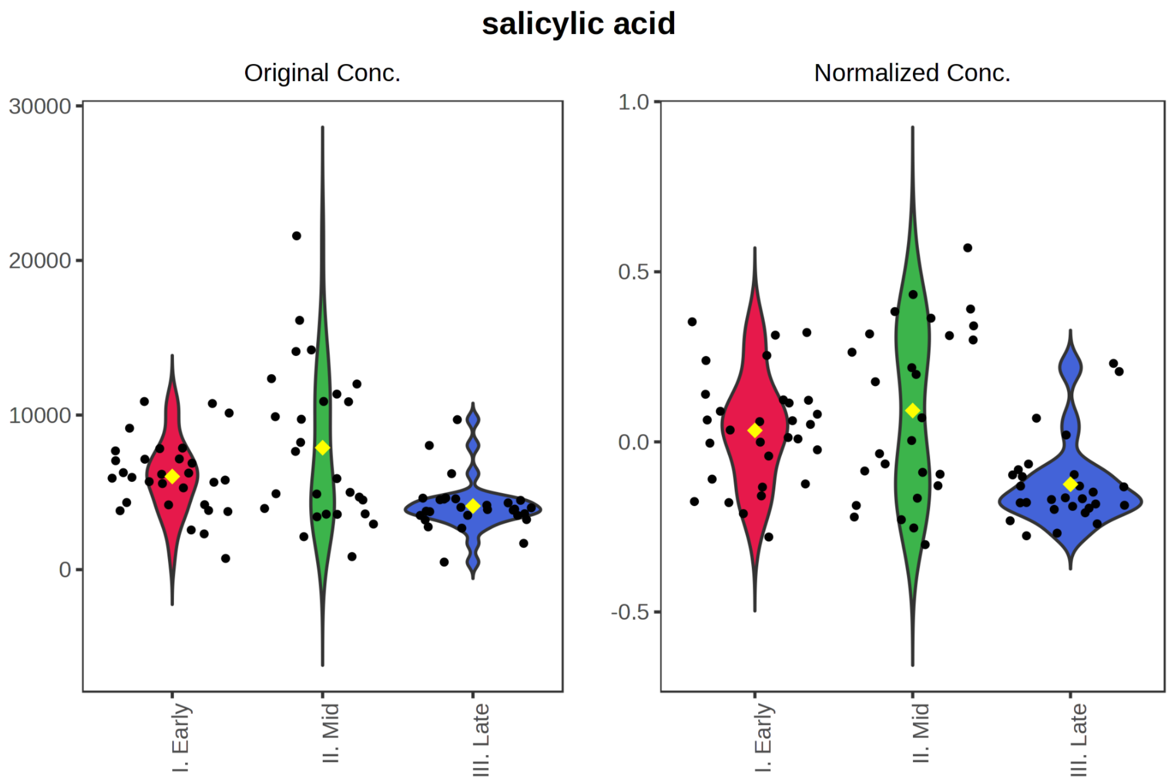
salicylic acid
Original Conc.
Normalized Conc.
30000
20000
10000
0
1.0
0.5
0.0
-0.5
I. Early
II. Mid
III. Late

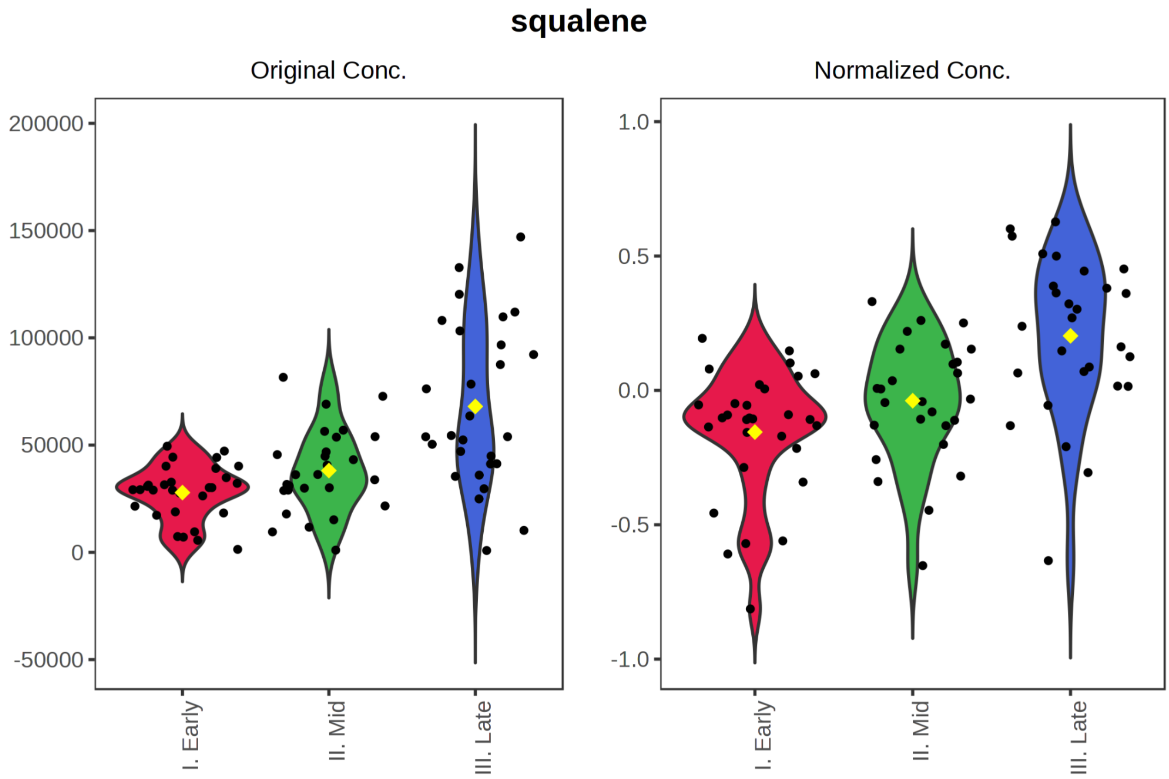
squalene
Original Conc.
Normalized Conc.
200000
150000
100000
50000
0
-50000
1.0
0.5
0.0
-0.5
-1.0
I. Early
II. Mid
III. Late

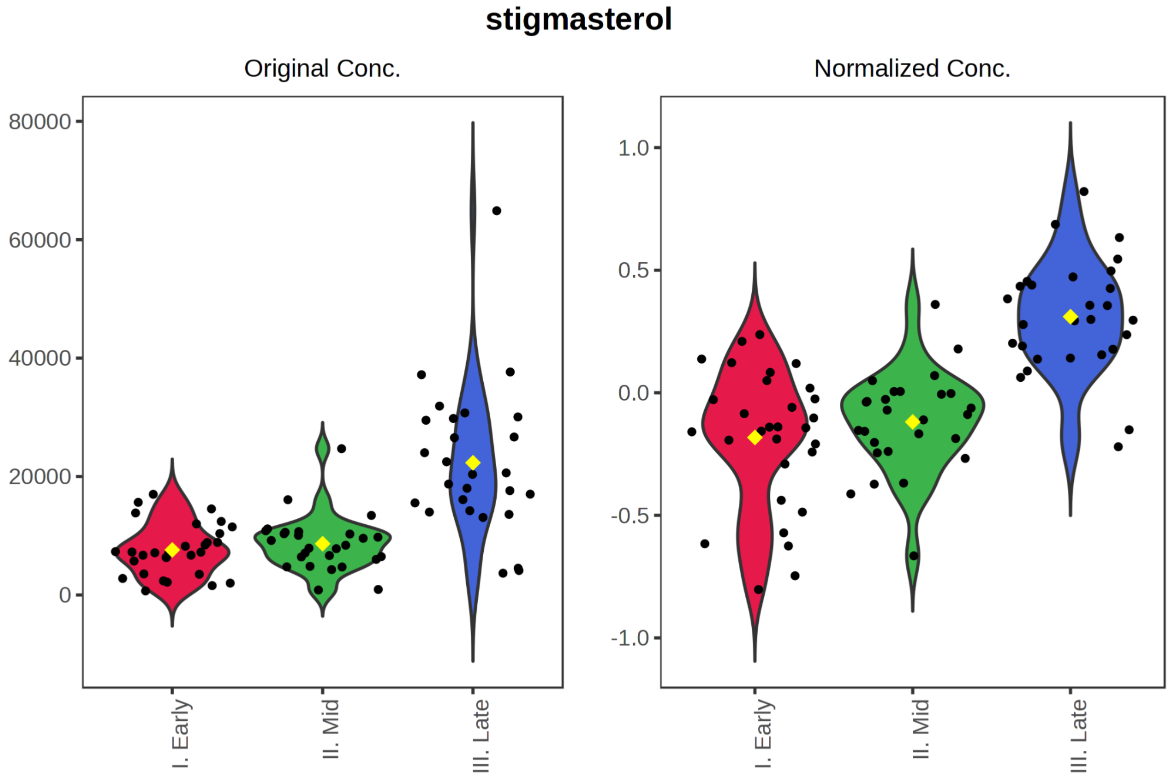
stigmasterol
Original Conc.
Normalized Conc.
80000
60000
40000
20000
0
1.0
0.5
0.0
-0.5
-1.0
I. Early
II. Mid
III. Late

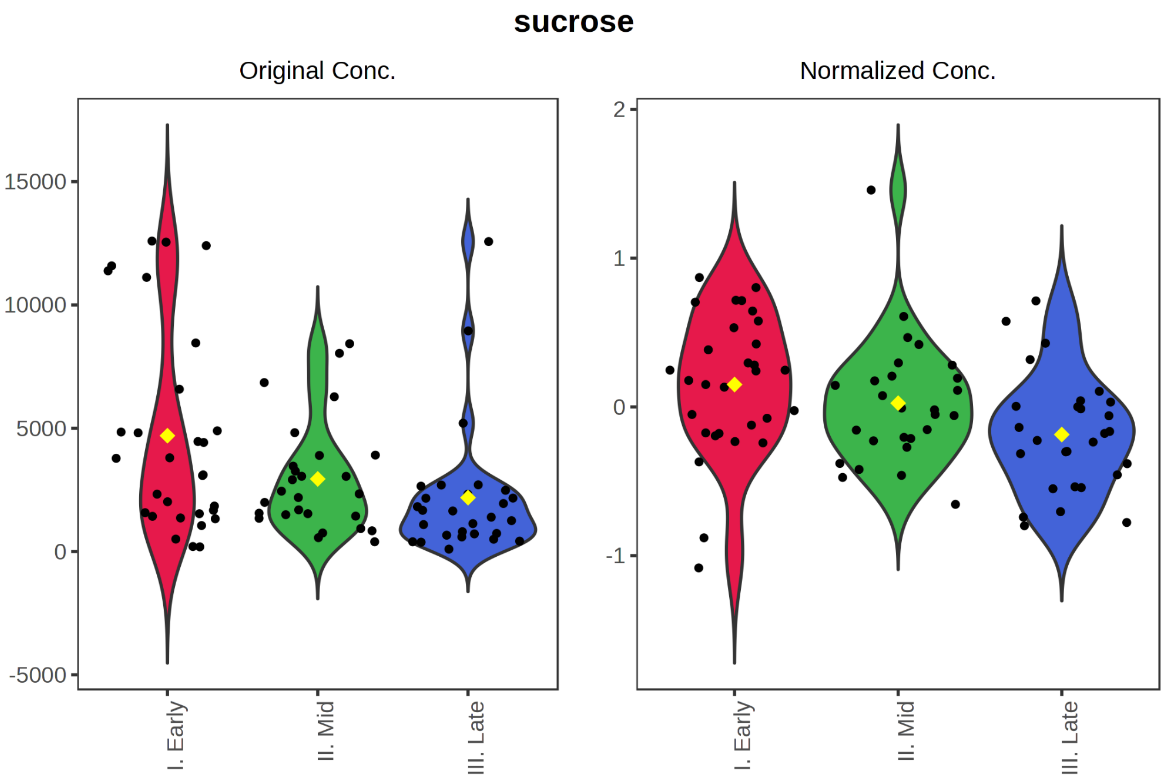
sucrose
Original Conc.
Normalized Conc.
15000
10000
5000
0
-5000
2
1
0
-1
I. Early
II. Mid
III. Late

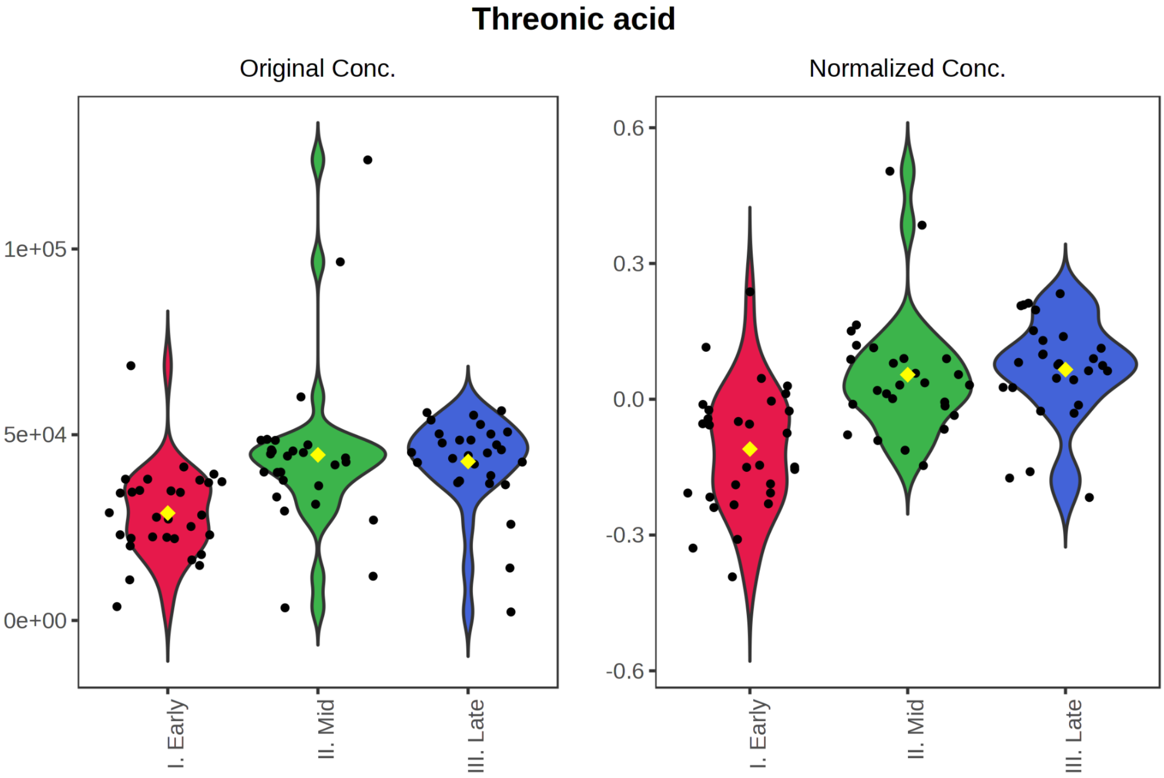
Threonic acid
Original Conc.
Normalized Conc.
1e+05
5e+04
0e+00
0.6
0.3
0.0
-0.3
-0.6
I. Early
II. Mid
III. Late

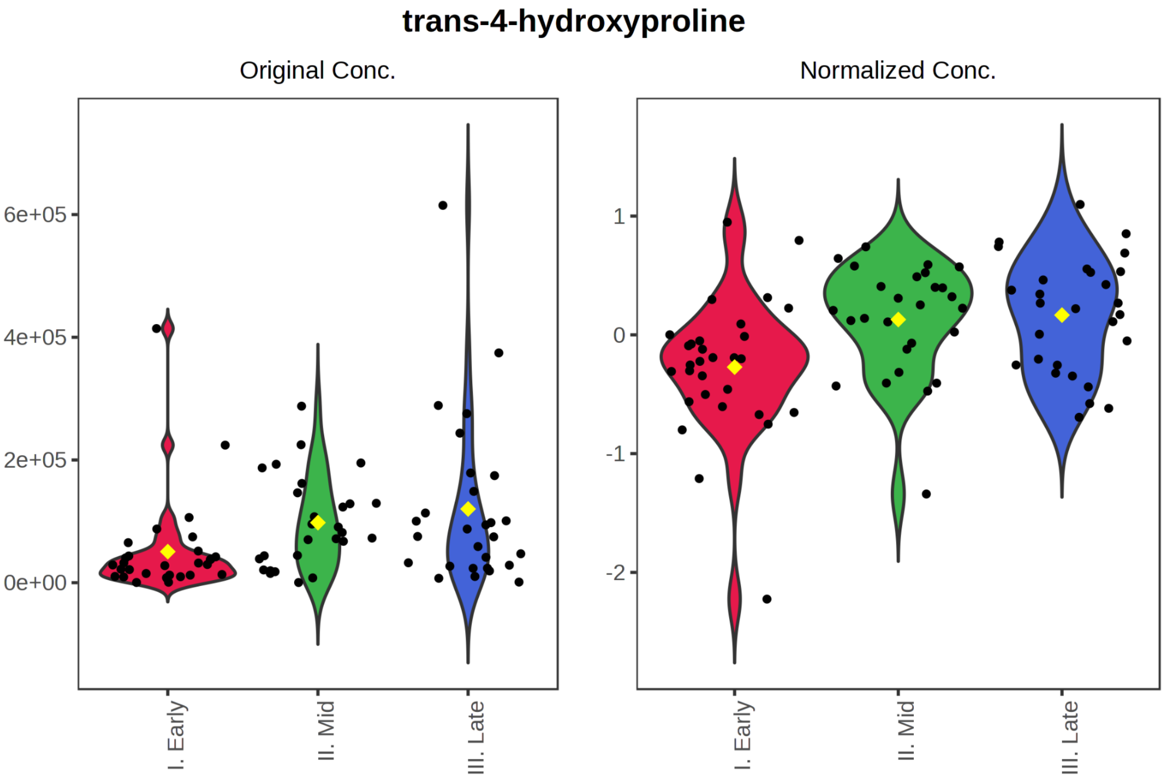
trans-4-hydroxyproline
Original Conc.
Normalized Conc.
6e+05
4e+05
2e+05
0e+00
1
0
-1
-2
I. Early
II. Mid
III. Late

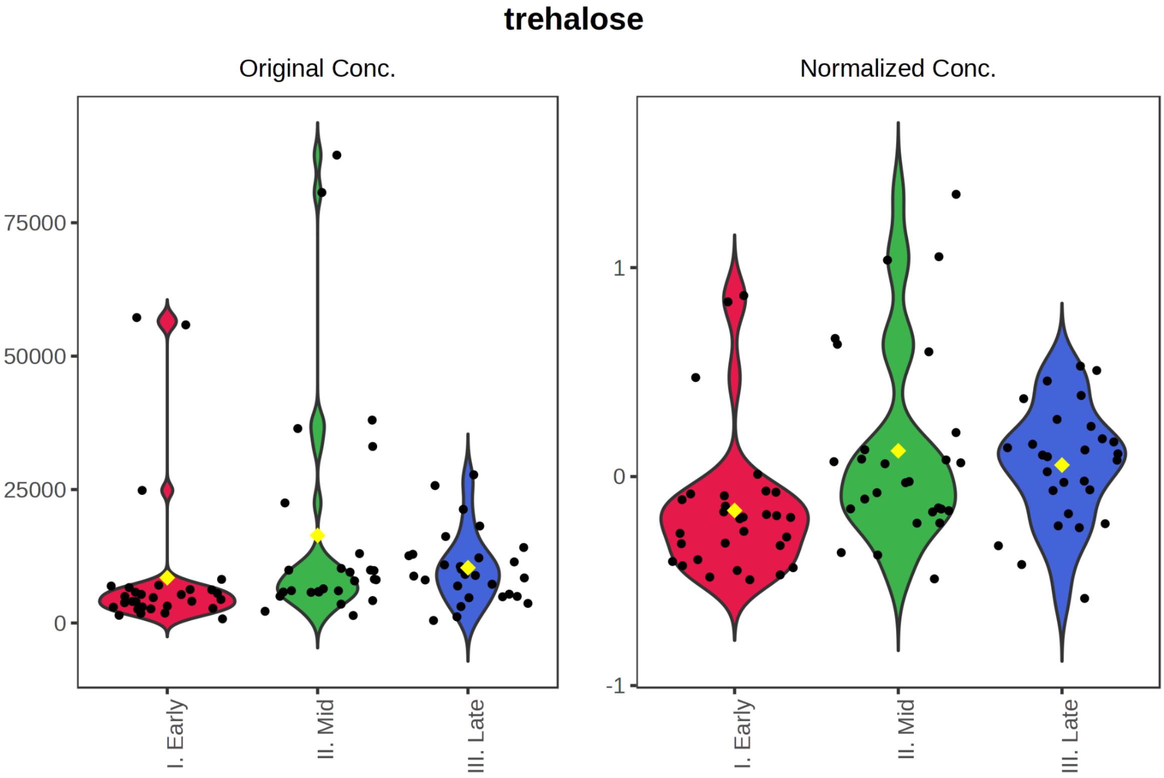
trehalose
Original Conc.
Normalized Conc.
75000
50000
25000
0
1
0
-1
I. Early
II. Mid
III. Late

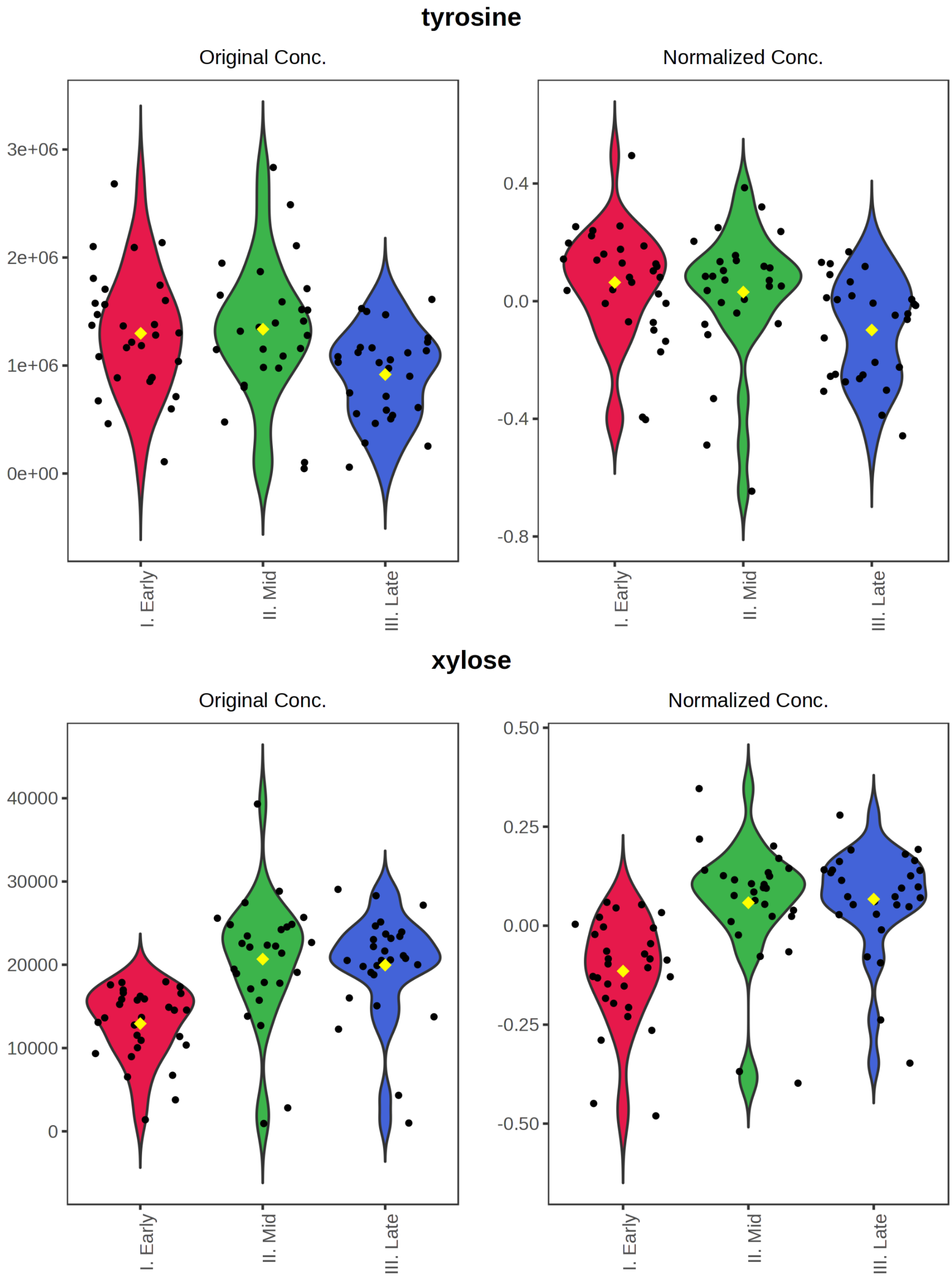
tyrosine
Original Conc.
Normalized Conc.
3e+06
2e+06
1e+06
0e+00
0.4
0.0
-0.4
-0.8
I. Early
II. Mid
III. Late
xylose
Original Conc.
Normalized Conc.
40000
30000
20000
10000
0
0.50
0.25
0.00
-0.25
-0.50
I. Early
II. Mid
III. Late

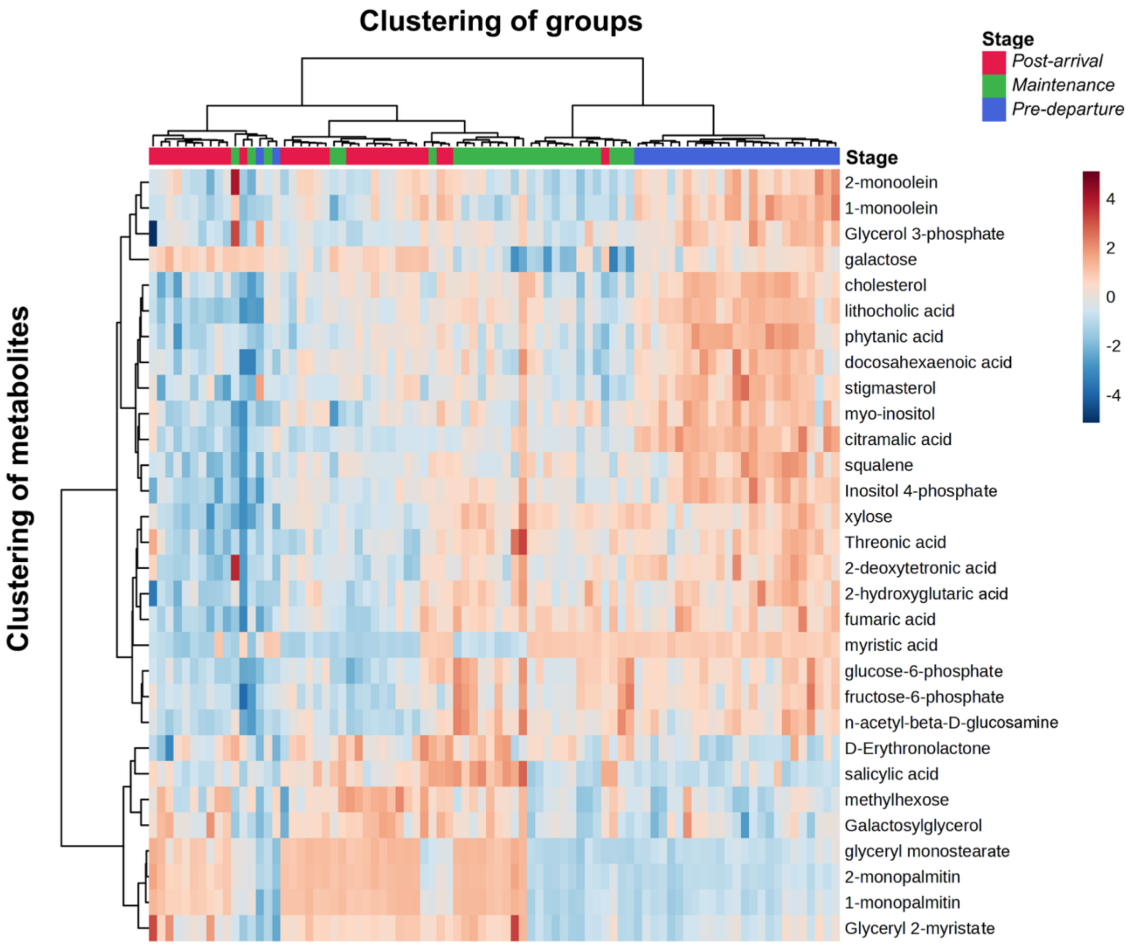

Supplementary Figure 3. Hierarchical Clustering Heatmaps for Top 30 plasma metabolites in Hudsonian godwits (*Limosa haemastica*), selected based on one-way ANOVA p-values across non-breeding stages. Groups were clustered using Euclidean distance with Ward clustering method. All biological replicates are shown (sample IDs are displayed at the bottom of each column). Normalized, log10 transformed, and mean scaled feature intensity values ranged from −4 to 4.

Supplementary Table 1. Statistically significant metabolites after FDR correction obtained from the two-way ANOVA.

Metabolite	stage(adj.p)	sex(adj.p)	Interaction(adj.p)
citramalic acid	**3.0456e-17**	0.96788	0.79712
2-monopalmitin	**3.7929e-10**	0.95819	0.79712
1-monopalmitin	**1.0152e-09**	0.96924	0.79712
stigmasterol	**3.6308e-09**	0.94196	0.98064
1-monoolein	**1.3184e-08**	0.96788	0.79712

myristic acid	**1.3184e-08**	0.96924	0.94301
phytanic acid	**1.1502e-07**	0.94196	0.79712
glyceryl monostearate	**1.2479e-07**	0.97391	0.79712
galactose	**9.9327e-07**	0.96788	0.92166
lithocholic acid	**1.1675e-06**	0.94196	0.79712
Glycerol 3-phosphate	**0,005076**	0.95819	0.79712
cholesterol	**9.6965e-06**	0.97391	0.79712
2-deoxytetronic acid	**9.6965e-06**	0.96765	0.79712
Threonic acid	**1.7524e-05**	0.96924	0.79712
Inositol 4-phosphate	**4.3522e-05**	0.78232	0.79712
xylose	**5.8427e-05**	0.78232	0.79712
n-acetyl-beta-D-glucosamine	**9.4553e-05**	0.97391	0.79712
2-hydroxyglutaric acid	**0.00010105**	0.78232	0.79712
fumaric acid	**0.00013432**	0.78232	0.90972
docosahexaenoic acid	**0.00019807**	0.97391	0.79712
Galactosylglycerol	**0.00019807**	0.81609	0.79712
2-monoolein	**0.00025124**	0.96788	0.79712
salicylic acid	**0.00028813**	0.95819	0.79712
methylhexose	**0.00030374**	0.78232	0.79712
squalene	**0.00039536**	0.705	0.987
fructose-6-phosphate	**0.00045445**	0.96788	0.79712
D-Erythronolactone	**0.00058918**	0.78232	0.90972
Glyceryl 2-myristate	**0.00058918**	0.95819	0.79712
myo-inositol	**0.00064179**	0.90606	0.90972
phosphate	**0.0016873**	0.78232	0.79712
glucose-6-phosphate	**0.0019012**	0.95819	0.79712
1,5-anhydroglucitol	**0.0026922**	0.94196	0.79712
glutamine	**0.0035499**	0.94196	0.90972
D-xylulose	**0.0035499**	0.96203	0.79712
N-acetylgalactosamine	**0.0036418**	0.96788	0.79712
ornithine	**0.0078333**	0.90606	0.91951
ascorbic acid	**0.011132**	0.78232	0.79712

trans-4-hydroxyproline	**0.011132**	0.96788	0.85699
glyceric acid	**0.020634**	0.78232	0.90972
maltose	**0.020634**	0.78232	0.79712
maltotriose	**0.020634**	0.97391	0.79712
oleic acid	**0.0235**	0.94196	0.89613
3-deoxyhexitol	**0.031333**	0.96765	0.9588
sucrose	**0.031333**	0.96788	0.91774
tyrosine	**0.031333**	0.95819	0.91522
creatinine	**0.036783**	0.81609	0.95421
trehalose	**0.0397**	0.96788	0.86167
hexitol	**0.041125**	0.96203	0.79712
fructose	**0.043163**	0.90606	0.79712
